# Total Synthesis of Aetokthonotoxin, the Cyanobacterial Neurotoxin Causing Vacuolar Myelinopathy

**DOI:** 10.1002/chem.202101848

**Published:** 2021-06-18

**Authors:** Manuel G. Ricardo, Markus Schwark, Dayma Llanes, Timo H. J. Niedermeyer, Bernhard Westermann

**Affiliations:** ^1^ Department of Bioorganic Chemistry Leibniz Institute of Plant Biochemistry Weinberg 3 06120 Halle/Saale Germany; ^2^ Department of Pharmaceutical Biology Martin-Luther-University Halle-Wittenberg Institute of Pharmacy/Pharmacognosy Hoher Weg 8 06120 Halle Germany; ^3^ present address: Department of Biomolecular Systems Max-Planck Institute of Colloids and Interfaces Am Mühlenberg 1 14476 Potsdam Germany

**Keywords:** biindole-coupling, bromination, indole, natural products, total synthesis

## Abstract

Aetokthonotoxin has recently been identified as the cyanobacterial neurotoxin causing Vacuolar Myelinopathy, a fatal neurologic disease, spreading through a trophic cascade and affecting birds of prey such as the bald eagle in the USA. Here, we describe the total synthesis of this specialized metabolite. The complex, highly brominated 1,2’‐biindole could be synthesized via a Somei‐type Michael reaction as key step. The optimised sequence yielded the natural product in five steps with an overall yield of 29 %.

## Graphical abstract

**Heterocyclic chemistry**: Synthesizing the eagle murder's weapon. With the identification of the alkaloid Aetokthonotoxin (AETX) as the neurotoxin causing wildlife deaths in the USA, further studies to characterize the biological effects of this natural product are mandatory. These studies will be enabled by a total synthesis of this natural product, which has been achieved in only five steps overall.

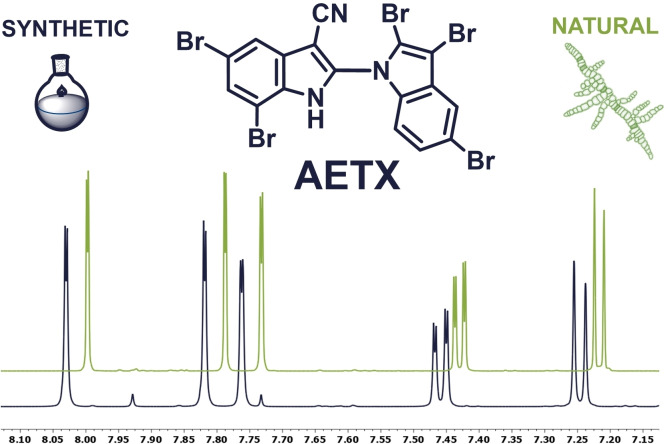

Aetokthonotoxin (AETX) is a pentabrominated biindole alkaloid produced by the cyanobacterium *Aetokthonos hydrillicola*, which grows epiphytically on an invasive plant, *Hydrilla verticillata*, in freshwater lakes.^[1]^ The toxin traverses the food chain from animals consuming the plant (and thus also the cyanobacterium) like waterfowl or snails and then on to raptors such as eagles (e. g. American bald eagle) and kites. While the mode‐of‐action of AETX is still unknown, it has been shown to elicit Vacuolar Myelinopathy in birds, which is characterized by a widespread vacuolization of the myelinated axons in the white matter of the brain and spinal cord of affected animals.^[1]^ AETX has also been found to be highly toxic to the nematode *C. elegans* (LC_50_ 40 nM),^[1]^ but susceptibility of mammals to this toxin has not been studied, yet.

In Figure 1, the structure of Aetokthonotoxin (**1**) is depicted, exhibiting several structural features rarely (if at all) found in natural products: i) AETX is the only known naturally produced 1,2’‐bi‐1*H*‐indole. ii) Only one other natural product contains an indole‐3‐carbonitrile.^[2]^ iii) Most prominent, also from the isotope pattern that is observed in mass spectrometry analyses, are the five bromo substituents accounting for 61 % of the molecular weight.

**Figure 1:**
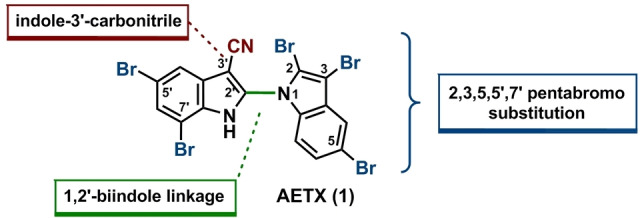
Structure of Aetokthonotoxin (AETX, 1) with structural features rarely found in natural products.

To enable detailed studies on the biological activity of this novel cyanotoxin, and thus to assess its potential impact on human health, a total synthetic route to **1** is a prerequisite. According to our retrosynthetic analysis (Scheme 1), the most obvious challenge is the coupling of two readily prepared indole subunits. Methods for this uncommon 1,2’‐biindole linkage have been explored in a very limited extent, using metal‐catalyzed ligation or nucleophilic displacement protocols.[^3^, ^4^, ^5^] Due to side‐reactions based on participation of the bromo‐phenylic moieties in these reactions, the use of such procedures seemed inappropriate for our target. Therefore, we devised that a Somei‐type Michael coupling fusing advanced indole intermediates could offer a straightforward alternative to access this 1,2’‐biindole.[^6^, ^7^, ^8^] This envisioned coupling would be the first example of nitrile‐modified Michael acceptors and extend the use of this method largely. In addition, in previous literature discussions, it was believed that this reaction could be a biomimetic approach to achieve this hitherto unknown indole‐linkage.^[5]^

**Scheme 1:**
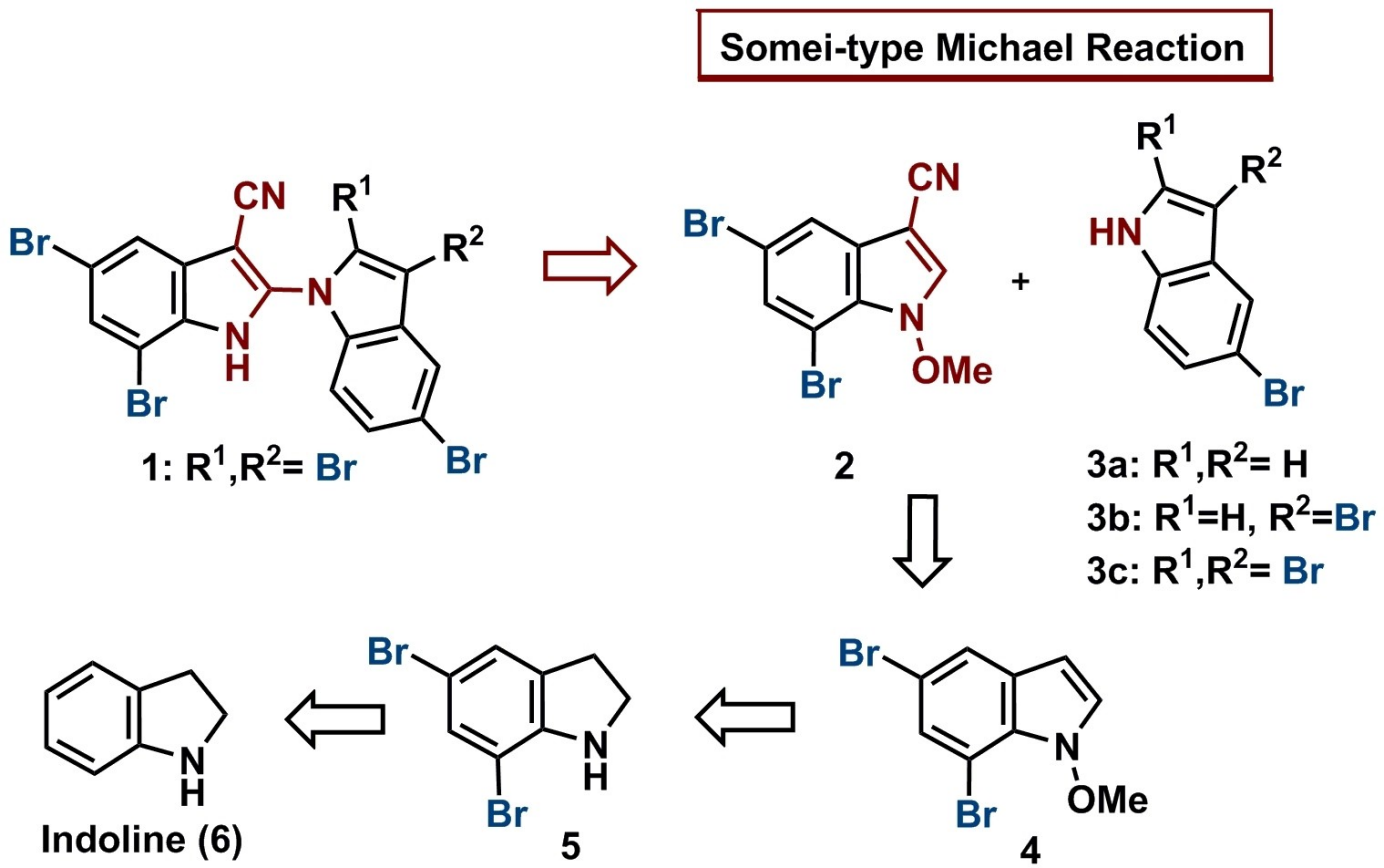
Retrosynthetic analysis of AETX (**1**).

Implementing the Somei coupling strategy, the two building blocks would have to be indoles **2** and **3 a**–**c** (Scheme 1). The key intermediate **2**, which holds the Michael‐acceptor reactivity (3‐cyano‐*N*‐methoxy substitution) demanded by the coupling process, was planned to be obtained either by indole synthesis starting from appropriate brominated precursors, or already functionalized indoles to be brominated subsequently. For the complementary Michael‐donors, the brominated indoles **3 a**–**c** were suggested, depending on the reactivity in the coupling reaction. While coupling with **3 c** would lead directly to the fully substituted natural product, coupling with **3 a**,**b** would require further bromination steps.

For the synthesis of the Michael acceptor **2**, we initially considered a protocol by Penoni et al. to be particularly convenient for the creation of *N*‐methoxy‐3‐substituted indoles.[^9^, ^10^] As depicted in Scheme 2 (strategy A), this approach consisted of the cycloaddition of nitroso benzenes with electron deficient alkynes affording desired indole **2**. Preceding the cycloaddition, 2,4‐dibromo aniline **7** was efficiently transformed into the nitroso derivative **8**.^[11]^ The cyano acetylene **9** was synthesized from commercially available propiol amide by dehydration (see Supporting Information).^[12]^ Conversely, attempts to use cyano acetylene **9** for the synthesis of **2** led to difficult to separate product mixtures after the cycloaddition reaction due to the formation of polymerized byproducts (see Supporting Information).

**Scheme 2:**
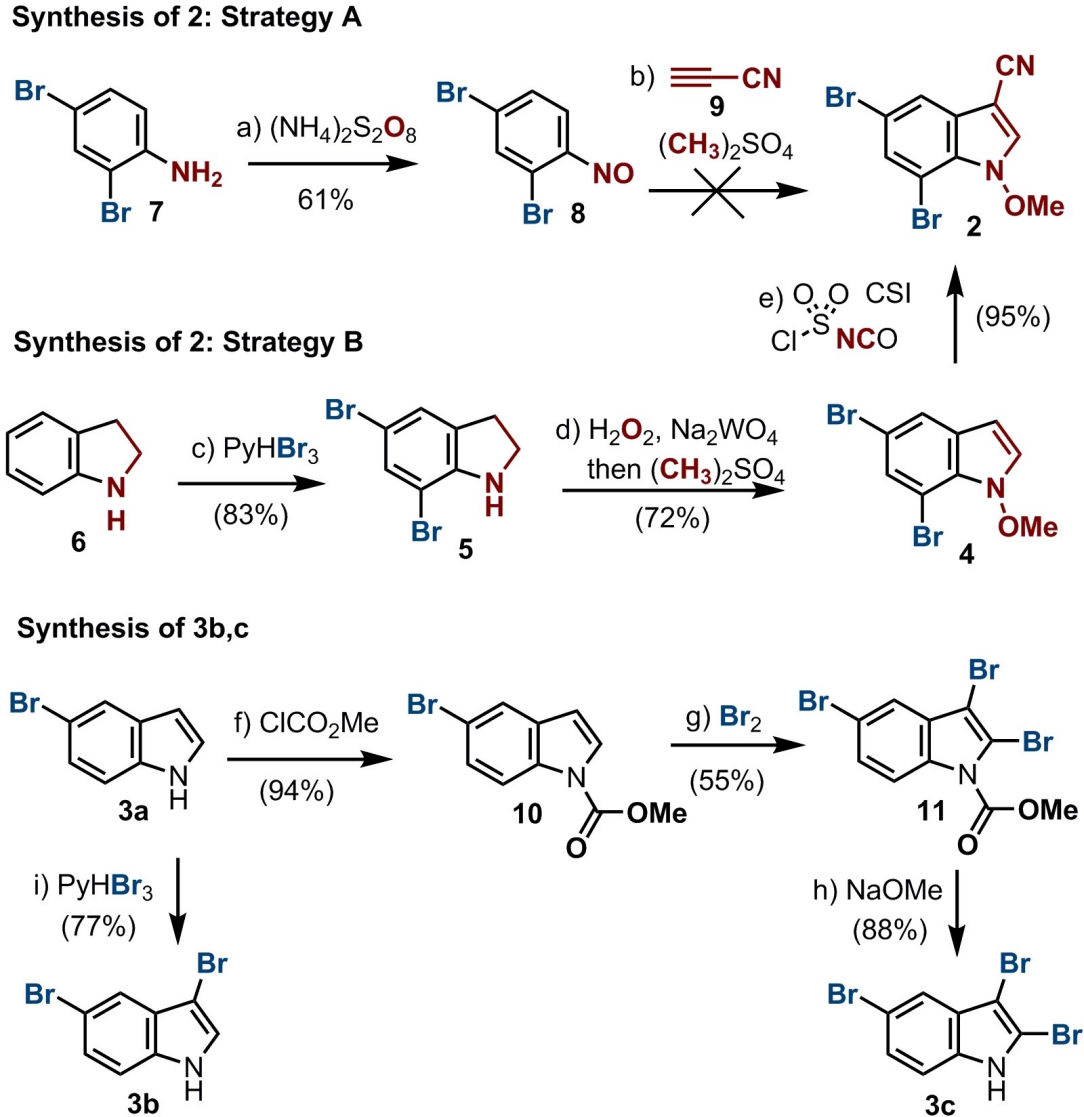
Synthesis of building blocks **2**, and **3 b**,**c**. Reagents and conditions: a) (NH_4_)_2_SO_4_ (5 equiv.), H_2_SO_4_ (5 equiv.), 0 °C, 4 h, 61 %; b) **9** (5 equiv.), K_2_CO_3_ (10 equiv.), (CH_3_)_2_SO_4_ (10 equiv.), toluene, 60 °C, 6 h, no reaction; c) PyHBr_3_ (2.2 equiv.), DCM, 48 h, 83 %; d) i: H_2_O_2_ (30 %, 10 equiv.), Na_2_WO_4_ (cat.), MeOH/H_2_O/THF 6 : 1 : 2, 0–5 °C, 1 h; ii: K_2_CO_3_ (3 equiv.), (CH_3_)_2_SO_4_, (4 equiv.), 0–5 °C, 1 h, 72 % (two steps); e) CSI (1 equiv.), MeCN, 0 °C, 1 h, then DMF, 0 °C, 1 h, 95 %; f) NaH (1.2 equiv.), ClCO_2_Me (1.2 equiv.), DMF, 0 °C, 2 h, 94 %; g) Br_2_ (8 equiv.), CCl_4_, r. t., 12 h, 55 %, h) NaH (1.5 equiv.), MeOH, reflux; 2 h, 88 %; i) PyHBr_3_ (1,1 equiv.), DCM, 12 h, 77 %.

A second alternative, depicted in Scheme 2 (strategy B), was envisioned to be based on the protocol developed by Somei et al. for the concerted oxidative transformation of indoline derivatives into *N*‐methoxy indoles.[^13^, ^14^, ^15^] Here, 5,7‐dibromoindoline **5** (available from indoline **6** in 83 % yield) is transformed to **4**, in 72 % yield following slightly modified protocols.^[14]^ Very gratifyingly, the procedure developed by Vorbrüggen et al. using CSI led to conversion of **4** to the wanted nitrile **2** in an isolated yield of 95 %.^[16]^ Overall, the synthesis of **2** could be achieved in gram scale in only three steps starting from commercially available educts.

In the best case, the synthesis of AETX (**1**) would have been achieved by coupling **2** with tribrominated indole **3 c** via intermediate **12** by an initial Michael‐reaction (Scheme 3). To achieve the 1,4‐C−N coupling, carbonyl moieties have been reported on the Micheal acceptor,^[14]^ here for the first time a cyano group is used. The biindole is restored by a loss of the methoxy moiety and rearomatization. The synthesis of this tribrominated indole, however, proved to be quite tedious, as most of the common bromination reagents gave only mixtures of multi‐brominated products of di (3,5; **3 b**), tri (2,3,5; **3 c**) and tetra (2,3,5,6)‐substituted bromoindoles. Thus, using bromine as bromination agent, **3 c** could be obtained in 45 % overall yield starting from **3 a** (Scheme 2).^[17]^ The synthesis of indole **3 b** has been evaluated following numerous established protocols, revealing that the method with PyHBr_3_ was most efficient in our hands (Scheme 2).[^18^, ^19^, ^20^]

**Scheme 3:**
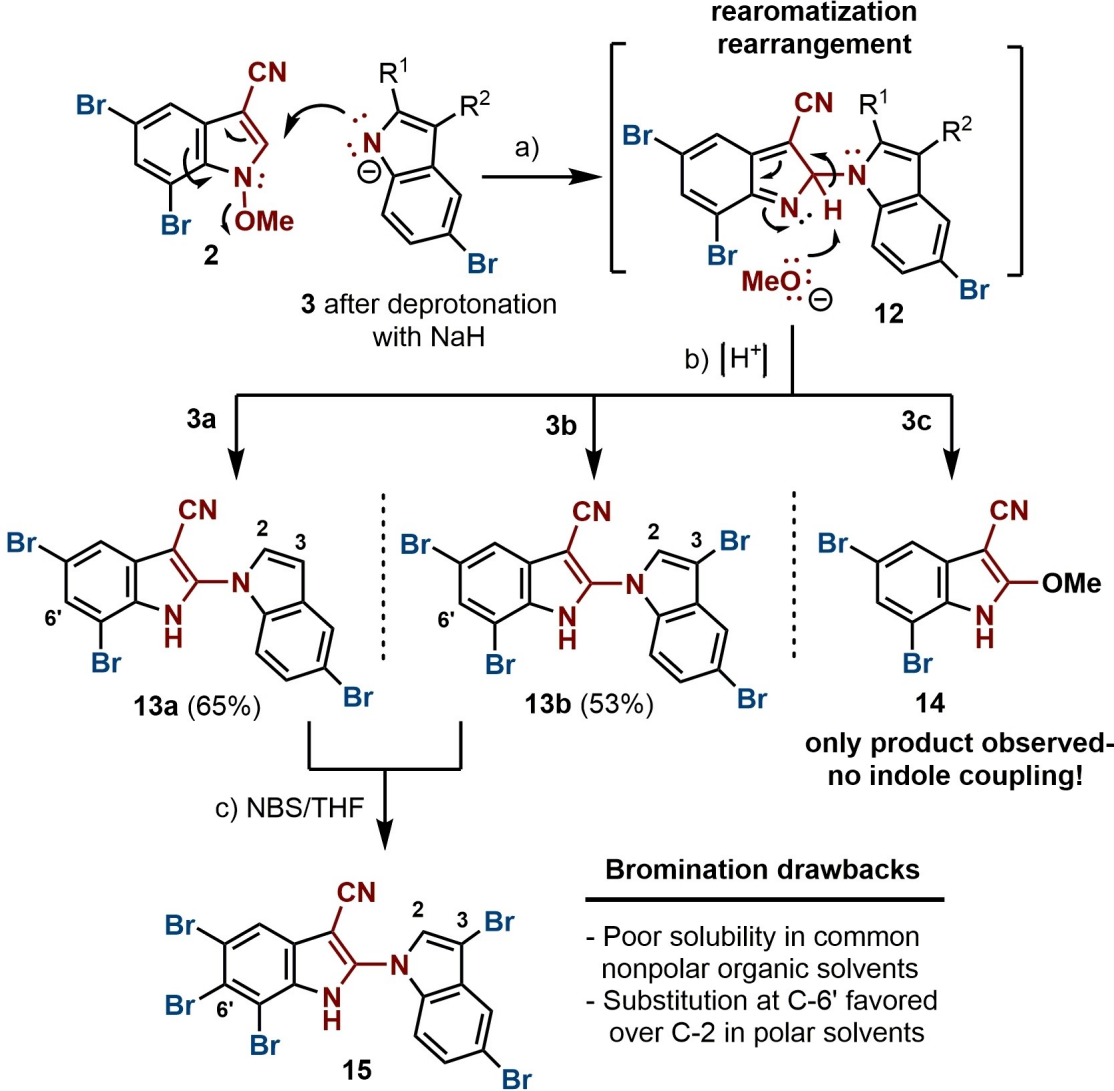
Somei‐Michael coupling attempts. Reagents and conditions: a) General conditions of Somei reaction: **3** (1.2 equiv.), NaH (1.1 equiv.), **2** (1 equiv.), DMF, 0 °C to r. t., 12 h; b) NH_4_Cl, **13 a** (65 %), **13 b** (53 %), for **3**c: **14** detected instead of biindole; c) All bromination attempts modify only C‐3 in **13 a** to form **13 b**, when NBS in THF are used, C‐6’ is then brominated to form **15**.

For the crucial biindole formation, the coupling step establishing the N1−C2’ linkage could not be achieved when using indole **3 c**; steric or stereoelectronic effects may be accountable for this. Different conditions were applied unsuccessfully, with the undesired 2‐methoxy‐3‐cyano‐5,7‐dibromoindole **14** being the only compound observed, as reported in the literature when poor nucleophiles are used (Scheme 3, Supporting Information).[^6^, ^8^]

Consequently, indoles **3 a** and **3 b** were employed in the coupling reactions. Considered as alternatives to the desired coupling, further bromination of the subsequent dimer would be necessary. The Somei couplings performed with mono‐brominated **3 a** and di‐brominated **3 b**, using NaH as base, confirmed the expected reactivity of the indole **2**, affording the desired N1−C2’ biindoles **13 a** and **13 b** in 65 % and 53 % yield, respectively (Scheme 3). With these results, the choice of using the Somei‐Michael reaction for the synthesis of AETX became a realistic strategy.

Having in hand advanced biindoles, lacking bromine atoms only at C‐2 (**13 b**) or C‐2 and C‐3 (**13 a**) with respect to the natural product; brominations at these most reactive positions were considered achievable due to methods already carried out in our studies (e. g. **11**).[^17^, ^19^, ^21^] However, the very poor solubility of **13 a**,**b** in most common organic solvents reduced the possibilities for the final bromination substantially, as the polarity of the solvent is reported to be crucial in the selectivity of these reactions.[^17^, ^19^] All bromination attempts, regardless of the agent used, did not afford **1** starting from **13 a**,**b** using THF or DMF as solvents. Position C‐3 of **13 a** was always smoothly brominated to form **13 b**, while the missing bromine substitution at C‐2 neither occurred nor took place at other positions rather than C‐2. We speculated that the electronic influence of the highly ionizable NH group (^1^H NMR signal over 12 ppm) along with a different puckering of the two indole units in such polar solvents would lead to form considerably electron enriched biindole species that guide the bromination to other, unwanted positions. The presence of a bromine atom at C‐6’ in a pentabromo biindole obtained in an experiment conducted with **13 a**, using NBS in THF, agreed with this hypothesis (Scheme 3).^[22]^

To extend the bromination alternatives, building block **2** was coupled with **3 a**, and subsequently, instead of acidic “work‐up”, intermediate **12** was quenched with SEM‐chloride to yield **16** in 74 % yield (Scheme 4). The SEM group was chosen as ideal protecting group to avoid interference of the NH‐proton in the required bromination steps and to boost hydrophobicity and, therefore, increase solubility and facilitate purification. According with our previous experiences in brominating procedures, we tried PyHBr_3_ in DCM first, observing a clean reaction to tetra‐brominated **17**. At this stage, attempts to selectively brominate at position C‐2 also failed. In further attempts, we turned our attention to DBDMH in DCE, as it was reported to achieve C‐2 and C‐3‐dibrominaton of indoles.^[17]^ To our delight, we were able, for the first time, to isolate a pentabromo biindole **18**. Subsequent SEM deprotection using 50 % TFA in DCM afforded the natural product **1**. Unfortunately, under these conditions, the yield was quite low. Optimization efforts yielded the final product in only 29 % despite full consumption of the starting material, as side reactions were observed. However, this result was inspiring, as it indicated the feasibility of the coupling/bromination strategy. Finally, applying 5 equivalents of Br_2_ in DCE,[^19^, ^23^, ^24^] a clean reaction could be obtained, leading to the satisfactory bromination at C‐2 and C‐3 at the same time. Most pleasingly, the reaction conditions also cleaved the SEM‐protecting group (triggered by HBr formed in situ) to afford **1** directly and in a one‐pot reaction. The bromination/deprotection sequence could be accomplished in 68 % yield. All spectroscopic and analytical data of synthesized AETX were in accordance with the isolated natural product.

**Scheme 4:**
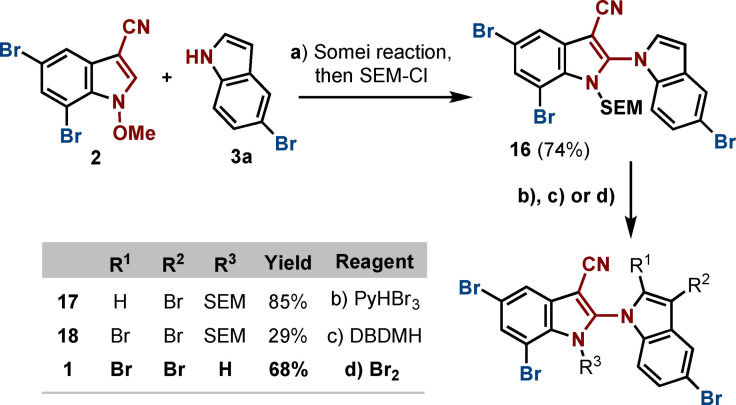
Synthesis of AETX (**1**). Reaction conditions: a) i: **3 a** (1.2 equiv.), NaH (1.1 equiv.), **2** (1 equiv.), DMF, 0 °C to r. t., 12 h; ii: SEM−Cl (2.5 equiv.), Cs_2_CO_3_, (2 equiv.), r. t., 12 h, 74 % (two steps); b) PyHBr_3_ (3 equiv.), DCM, 12 h, 85 % (**17**); c) DBDMH (3 equiv.), DCE, reflux, 12 h, 29 % (**18**); d) Br_2_ (5 equiv.), DCE, r. t., 1 h, 68 % (**1**).

In summary, we could achieve the first total synthesis of Aetokthonotoxin in five steps with an overall yield of 29 %. The crucial biindole‐linkage could be realized via a Somei‐Michael reaction, the protection/deprotection reactions were both carried out on the fly as quenching reactions. The highly convergent and flexible synthesis presented here may facilitate remarkable opportunities for further biological studies upon derivatization of this alkaloid.

## Conflict of interest

The authors declare no conflict of interest.

## Supporting information

As a service to our authors and readers, this journal provides supporting information supplied by the authors. Such materials are peer reviewed and may be re‐organized for online delivery, but are not copy‐edited or typeset. Technical support issues arising from supporting information (other than missing files) should be addressed to the authors.

Supporting Information
