# Supplementary material for: Total Synthesis of Aetokthonotoxin, the Cyanobacterial Neurotoxin Causing Vacuolar Myelinopathy
Source: Chemistry. 2021 Jun 18;27(47):12032–5. doi: 10.1002/chem.202101848 (PMC8453946; doi:10.1002/chem.202101848)
Supplement: Supplementary file 1 — Supporting Information [file CHEM-27-12032-s001.pdf]

Chemistry–A European Journal

Supporting Information

Total Synthesis of Aetokthonotoxin, the Cyanobacterial Neurotoxin Causing Vacuolar Myelinopathy

Manuel G. Ricardo,* Markus Schwark, Dayma Llanes, Timo H. J. Niedermeyer, and Bernhard Westermann*

Author Contributions

B.W. Conceptualization:Equal; Data curation:Equal; Formal analysis:Equal; Project administration:Lead; Resources:Equal; Supervision:Lead; Writing – original draft:Lead; Writing – review & editing:Lead
M.R. Conceptualization:Equal; Data curation:Lead; Formal analysis:Lead; Investigation:Lead; Methodology:Lead; Writing – review & editing:Lead
M.S. Data curation:Supporting; Formal analysis:Supporting; Investigation:Supporting; Methodology:Supporting; Writing – review & editing:Supporting
D.L. Data curation:Supporting; Formal analysis:Supporting; Investigation:Supporting; Methodology:Supporting
T.N. Conceptualization:Supporting; Data curation:Supporting; Formal analysis:Supporting; Funding acquisition:Equal; Project administration:Supporting; Resources:Supporting; Writing – review & editing:Supporting

Table of contents

Abbreviations 2

General information 2

Synthesis and characterization of building blocks 3

Synthesis and characterization of biindoles 19

Bromination of biindoles 26

Abbreviations

AcOEt, ethyl acetate; AETX, Aetokthonotoxin; APT, Attached Proton Test; CSI, chlorosulfonyl isocyanate; DBDMH, 1,3-dibromo-5,5-dimethylhydantoin; DCE, dichloroethane; DCM, dichloromethane; DMF, dimethylformamide; DMSO, dimethyl sulfoxide; ESI-MS, electrospray ionization mass spectrometry; HR-MS, high resolution mass spectrometry; J, coupling constant; MeCN, acetonitrile; MeOH, methanol; NBS, *N*-bromosuccinimide; NMR, nuclear magnetic resonance; Py, pyridine; Rf, retention factor; RT, room temperature; SEM, trimethylsilylethoxymethyl, TFA, trifluoroacetic acid; THF, tetrahydrofuran; TLC, thin layer chromatography; TMS, tetramethylsilane.

General information

All starting materials were purchased from commercial sources and used without further purification. ^{1}H NMR and ^{13}C NMR spectra were recorded in a Varian Mercury 400 NMR spectrometer at 399.94 MHz and 100.57 MHz, respectively. Chemical shifts (δ) are reported in ppm relative to the TMS (^{1}H NMR) or to the solvent signal (^{13}C NMR). The negative-ion high-resolution ESI mass spectra were obtained with an Orbitrap Elite mass spectrometer (Thermo Fisher Scientific, Germany) equipped with an HESI electrospray ion source (negative spray voltage 3.5 kV, capillary temperature 275 °C, source heater temperature 250 °C, FTMS resolution 30000). A TripleToF 6600-1 mass spectrometer (Sciex) was also used for high-resolution mass spectrometry, which was equipped with an ESI-DuoSpray-Ion-Source (operating in positive ion mode) and controlled by Analyst 1.7.1 TF software (Sciex). The ESI source operation parameters were as follows: ion spray voltage: 5.500 V, nebulizing gas: 60 p.s.i., source temperature: 450 °C, drying gas: 70 p.s.i., curtain gas: 35 p.s.i. Data acquisition was performed in the MS1-ToF mode, scanned from 100 to 1500 Da with an accumulation time of 50 ms. Melting points were determined with a Leica DM LS2 microscope.

Synthesis and characterization of building blocks

1. Synthesis of 5,7-dibromo-1-methoxy-1*H*-indole-3-carbonitrile (2)

Strategy A: Via "Penoni" cycloaddition (failed attempt)

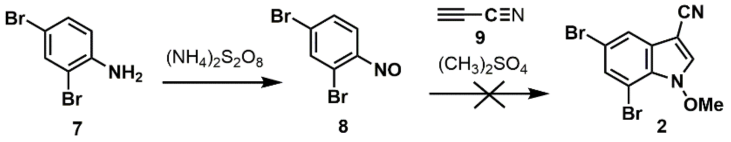

Synthesis of 2,4-dibromo-1-nitrosobenzene (7)

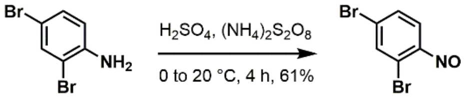

Following a reported protocol,[1] ammonium persulfate (4.6 g, 0.02 mol) and sulfuric acid (6.6 g, 0.067 mol) are stirred at room temperature for 1 h. This mixture is poured into 40 g of ice, after which 120 ml of water are added, followed by 2,4-dibromoaniline **7** (1.0 g, 0.04 mol). The mixture is allowed to reach room temperature and stirred for 4 h. The resulted precipitate is filtered off, washed with water, and dried in a desiccator to afford pure 2,4-dibromo nitrosobenzene **8** (0.65 g, 61%) as a brown solid. ^{1}H NMR (400 MHz, $CDCl_3$): δ = 8.20 (d, *J* = 1.9 Hz, 1H, H-3), 7.44 (dd, *J* = 8.4, 1.7 Hz, 1H, H-5), 6.10 (d, *J* = 8.6 Hz, 1H, H-6). ^{13}C NMR (101 MHz, $CDCl_3$): δ = 159.6 (C-1), 137.5 (C-5), 133.7 (C-4), 132.1 (C-2), 130.8 (C-3), 110.0 (C-6).

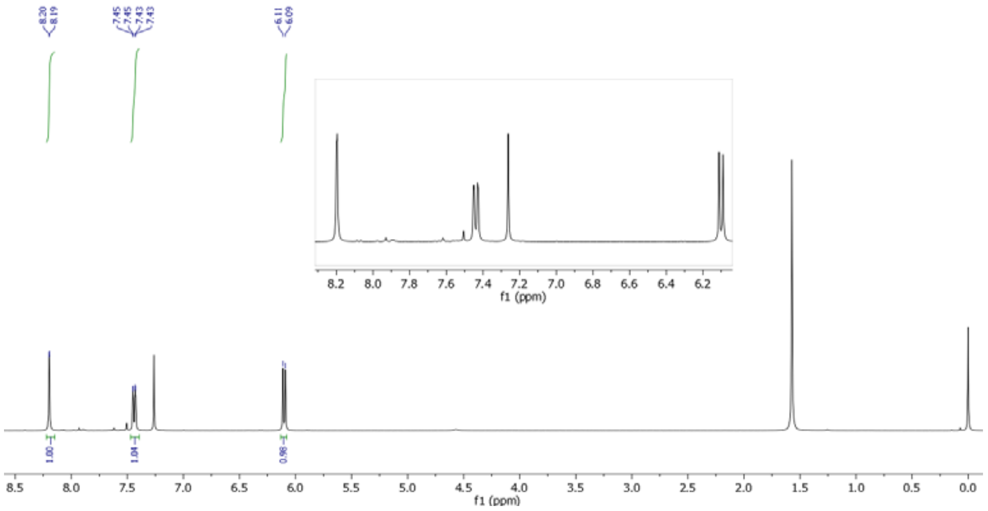

Figure S1. ^{1}H NMR (400 MHz, $CDCl_3$) spectrum of 2,4-dibromo-1-nitrosobenzene (**8**).

[1] A. M. Churakov, O. Y. Smirnov, S. L. Ioffe, Y. A. Strelenko, V. A. Tartakovsky, *Russ. Chem. Bull.* **1994**, *43*, 1532–1535

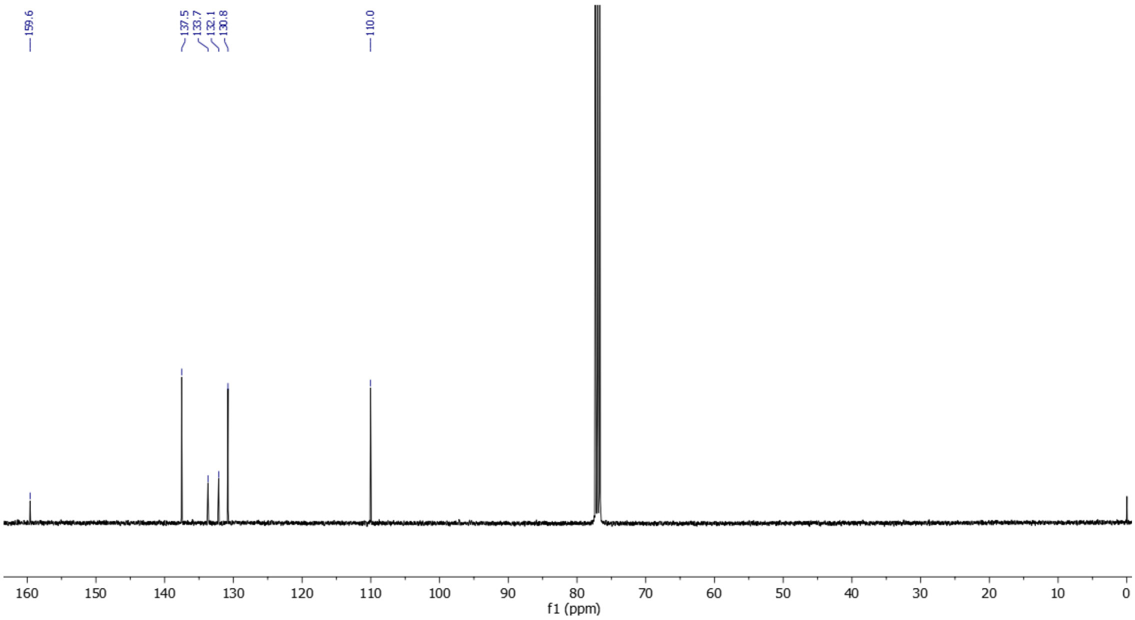

Figure S2. ^{13}C NMR (101 MHz, $CDCl_3$) spectrum of 2,4-dibromo-1-nitrosobenzene (**8**).

Synthesis of propiolonitrile (9)

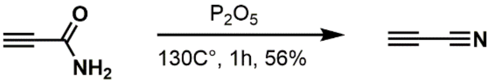

Propiolamide (1.0 g, 14.5 mmol) and P_2O_5 (8.2 g, 5.8 mmol) are carefully mixed and heated to 130 °C, as described in the literature.[2] Propiolonitrile **9** (0.41 g, 56%) is collected by distillation as a white solid at -78 °C. *Caution: This product is a severe vesicant, it penetrates globes and causes painful burns and blistering!* ^{1}H NMR (400 MHz, $CDCl_3$): δ = 2.52 (s, 1H). ^{13}C NMR (101 MHz, $CDCl_3$): δ = 104.5 (*C*N), 72.9 (CH), 57.6 (C).

[2] R. J. Halter, R. L. Fimmen, R. J. McMahon, S. A. Peebles, R. L. Kuczkowski, J. F. Stanton, *J. Am. Chem. Soc.* **2001**, *123*, 12353–12363.

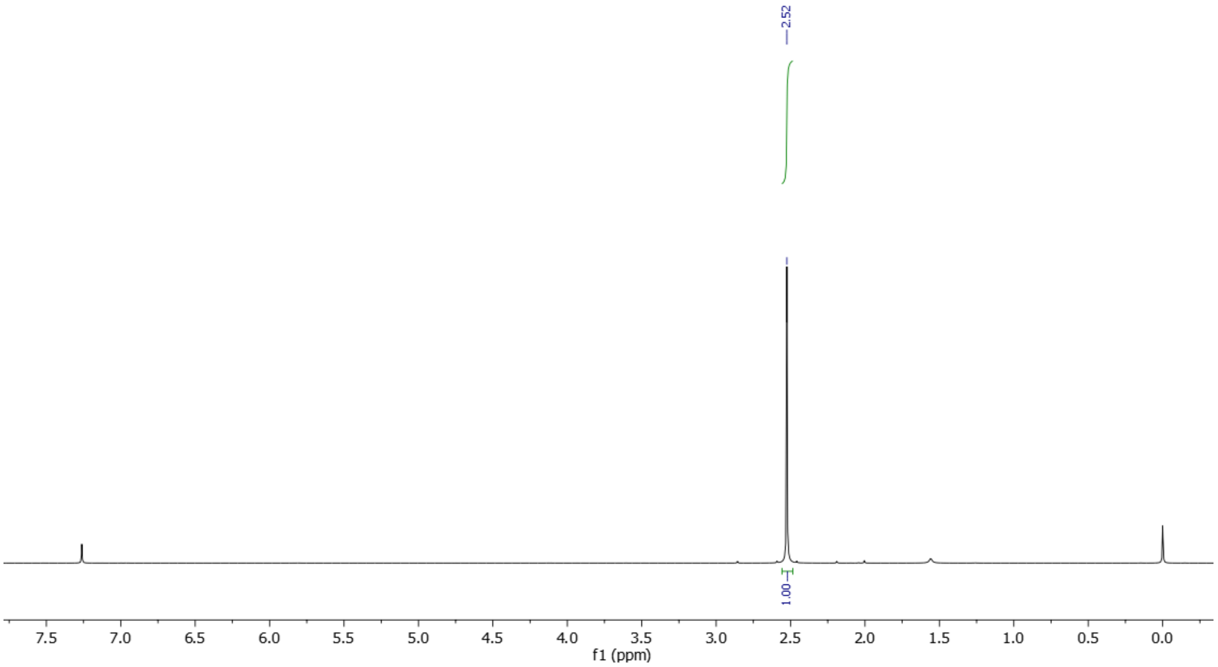

Figure S3. ^1H NMR (400 MHz, $CDCl_3$) spectrum of propiolonitrile (**9**).

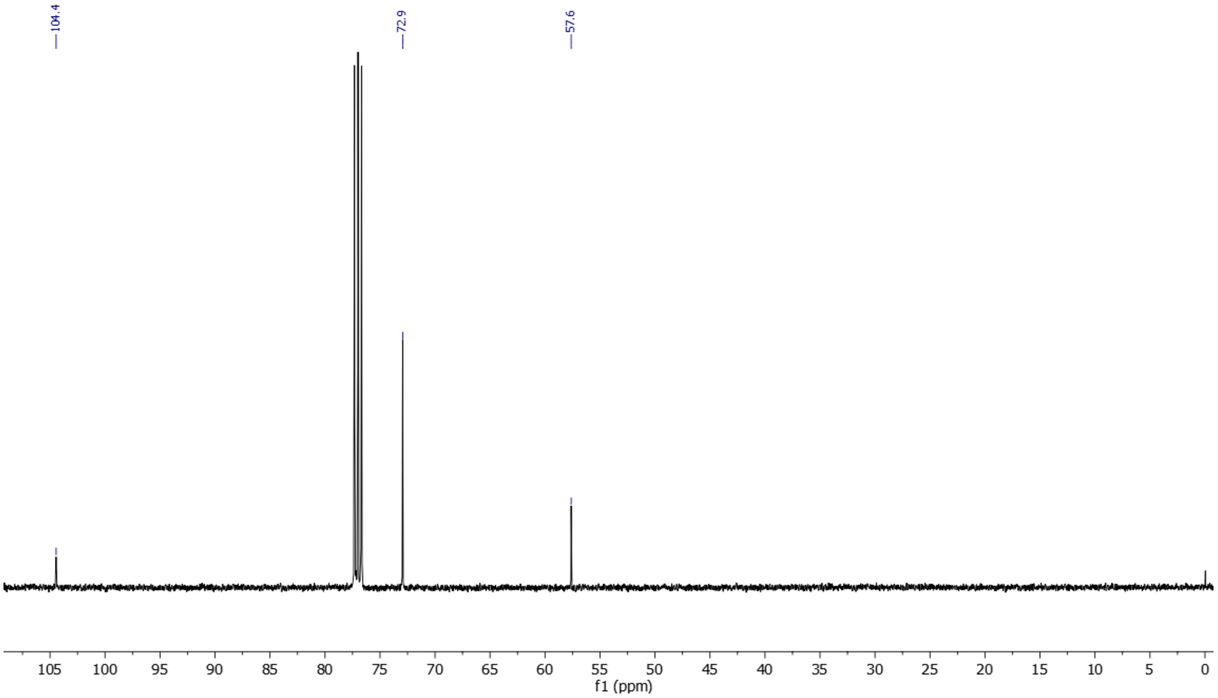

Figure S4. ^{13}C NMR (101 MHz, $CDCl_3$) spectrum of propiolonitrile (**9**).

Attempts to "Penoni" indole synthesis with 2,4-dibromo-1-nitrosobenzene (7)

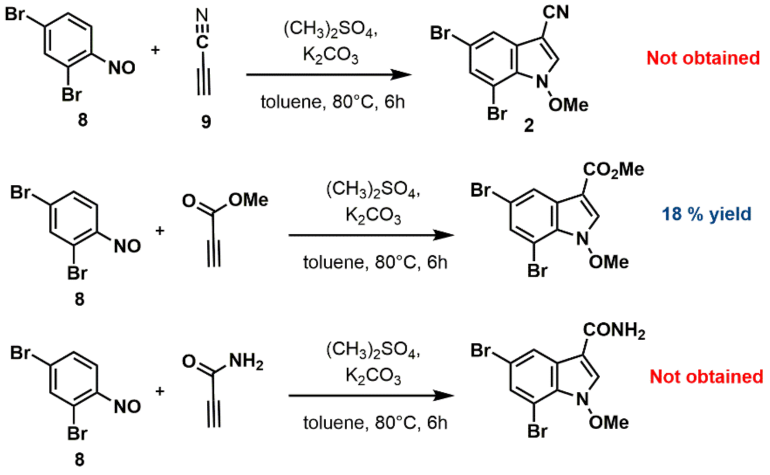

The Penoni cycloaddition has been extensively explored for the construction of *N*-OMe indoles using various aryl alkynes in moderate to excellent yields.[3] The use of alkynes with electron withdrawing groups has been limited to methyl propiolate only. Therefore, we envisioned propiolonitrile **9** could be used for the cycloaddition with brominated nitrosobenzenes and generate in only one step the desired 5,7-dibromo-1-methoxy-1H-indole-3-carbonitrile **2**. Thus, 2,4-dibromo-1-nitrosobenzene **8** (100 mg, 0.38 mmol) was reacted with propiolonitrile **9** (385 mg, 7.55 mmol) in the presence of K_2CO_3 (312 mg, 2.3 mmol) and $(CH_3)_2SO_4$ (191 µL, 2.3 mmol) according to the conditions described.[3] Unfortunately, a very complex mixture of products was obtained with no sign of the desired product. Changing the reaction conditions by using different solvents, higher temperatures or even microwave heating, did not show any improvement. While methyl propiolate or propiolamide have been reported to generate the corresponding indole derivative,[3] the nitrile functionality can be obtained by functional group transformations thereof, subsequently. However, none of these alkynes efficiently reacted with the 2,4-dibromo-1-nitrosobenzene **8**. In the reaction with propiolamide, there was no sign of product formation. Only with methyl propiolate, the cycloaddition was achieved in 18% yield (NMR of the cycloaddition product detailed as follow). With these results, the present strategy was discarded. ^{1}H NMR (400 MHz,$CDCl_3$): δ = 8.30 (d, *J* = 1.7 Hz, 1H, H-4), 7.95 (s, 1H, H-2), 7.59 (d, *J* = 1.7 Hz, 1H, H-6), 4.15 (s, 3H, N-OC*H*$_3$), 3.91 (s, 3H, CO_2C*H*$_3$).^{13}C NMR (101 MHz, $CDCl_3$): δ = 163.9

[3] a) A. Penoni, G. Palmisano, G. Broggini, A. Kadowaki, K. M. Nicholas, *J. Org. Chem.* **2006**, *71*, 823–825. b) G. Ieronimo, A. Mondelli, F. Tibiletti, A. Maspero, G. Palmisano, S. Galli, S. Tollari, N. Masciocchi, K. M. Nicholas, S. Tagliapietra, G. Cravotto, A. Penoni, *Tetrahedron* **2013**, *69*, 10906–10920.

(CO_2CH_3), 131.1 (C-2), 130.4 (C-6), 128.5 (C-7a), 126.3 (C-3), 123.5 (C-4), 115.8 (C-5), 103.3 (C-3a), 102.7 (C-7), 68.5 (N-OCH_3), 51.4 (CO_2CH_3).

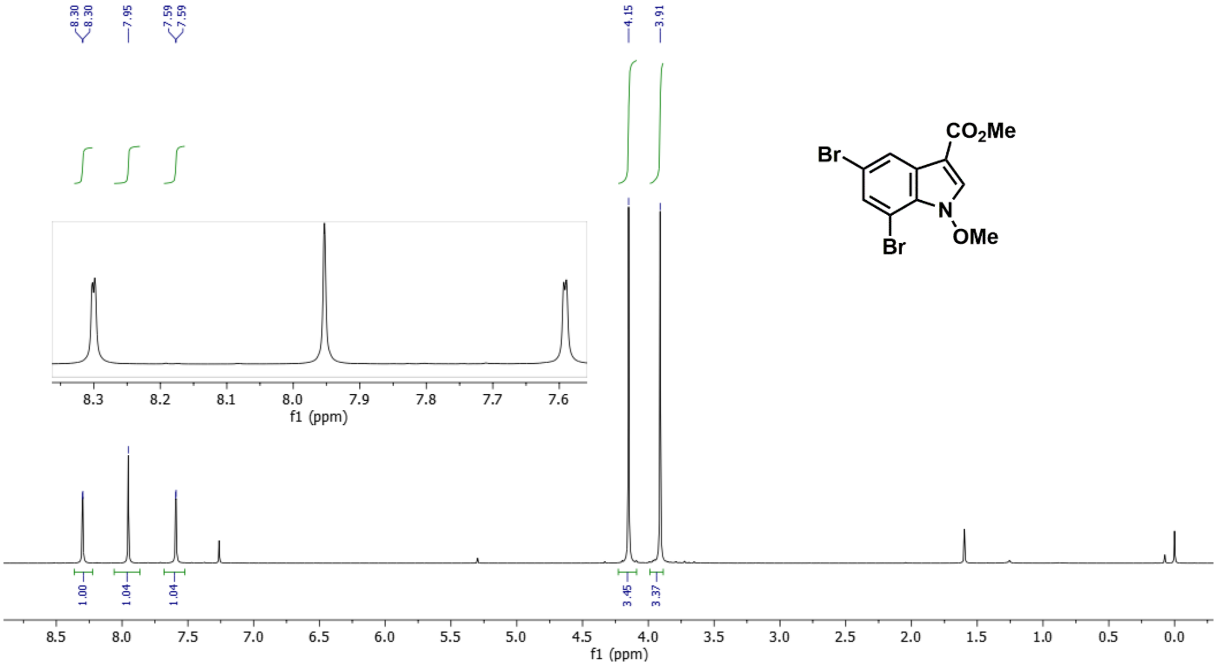

Figure S5. ^{1}H NMR (400 MHz, $CDCl_3$) spectrum of methyl 5,7-dibromo-1-methoxy-1*H*-indole-3-carboxylate.

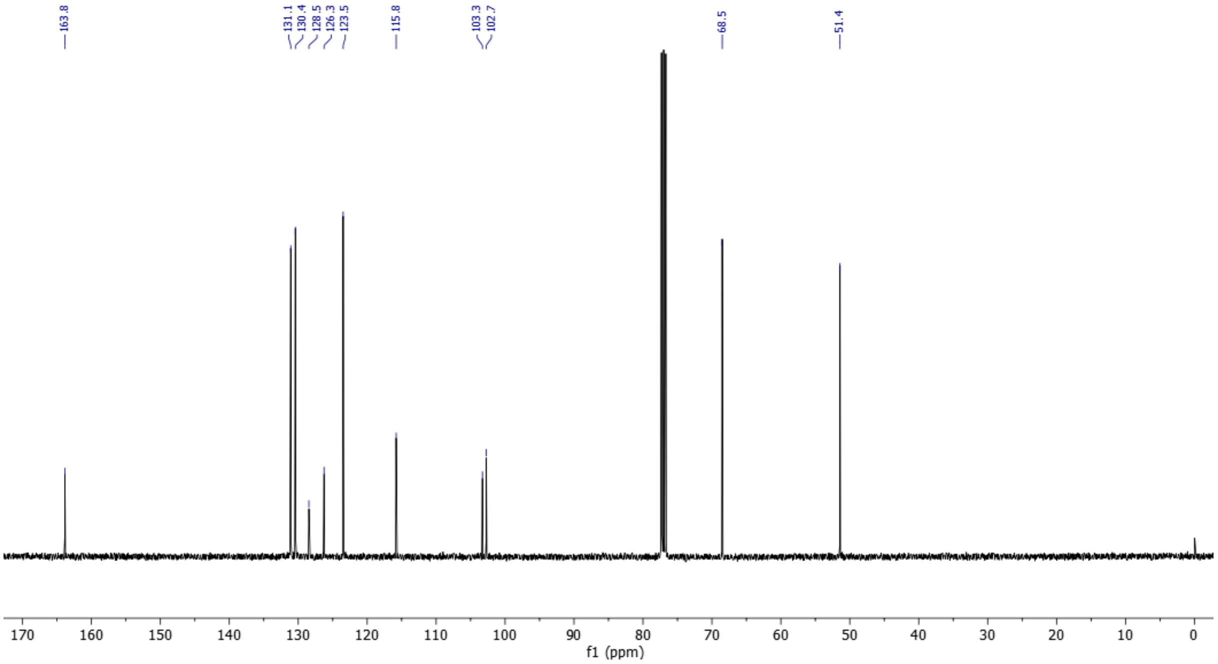

Figure S6. ^{13}C NMR (101 MHz, $CDCl_3$) spectrum of methyl 5,7-dibromo-1-methoxy-1*H*-indole-3-carboxylate.

Strategy B: Via "Somei" oxidation of indolines

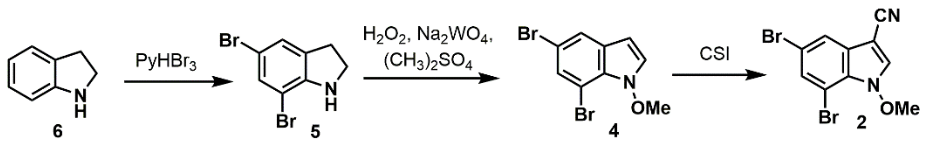

Synthesis of 5,7-dibromoindoline (5)

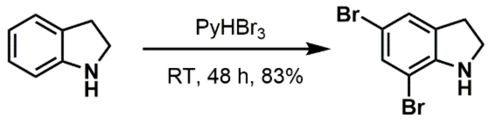

Indoline (2.0 g, 0.017 mol) is dissolved in 60 mL of DCM, $PyHBr_3$ (11.8 g, 0.037 mol) is added, and the reaction mixture is stirred for 48 h at room temperature. The crude is diluted with 50 mL of chloroform and washed successively with sodium bisulfate (10%), sodium bicarbonate (sat. soln.), and brine. The organic phase is concentrated at reduced pressure and purified by column chromatography (n-hex/EtOAc 10:1) to afford the 2,4-dibromoindoline (4.6 g, 83%) as purple syrup. R*f* (n-hex/EtOAc 4:1) = 0.52. ^{1}H NMR (400 MHz, $CDCl_3$): δ = 7.28 (d, *J* = 1.8 Hz, 1H, H-6), 7.11 (d, *J* = 1.6 Hz, 1H, H-4), 3.99 (s, 1H, NH), 3.62 (t, *J* = 8.5 Hz, 2H, H-2), 3.13 (t, *J* = 8.5 Hz, 2H, H-3). ^{13}C NMR (101 MHz, $CDCl_3$): δ = 149.4 (C-7a), 132.2 (C-3a), 131.7 (C-6), 126.5 (C-4), 109.6 (C-5), 103.0 (C-7), 46.9 (C-2), 30.7 (C-3). ESI-MS: m/z = 275.5 $[M+H]^+$; calcd. for $C_8H_8N^{79}Br_2$: 275.9.

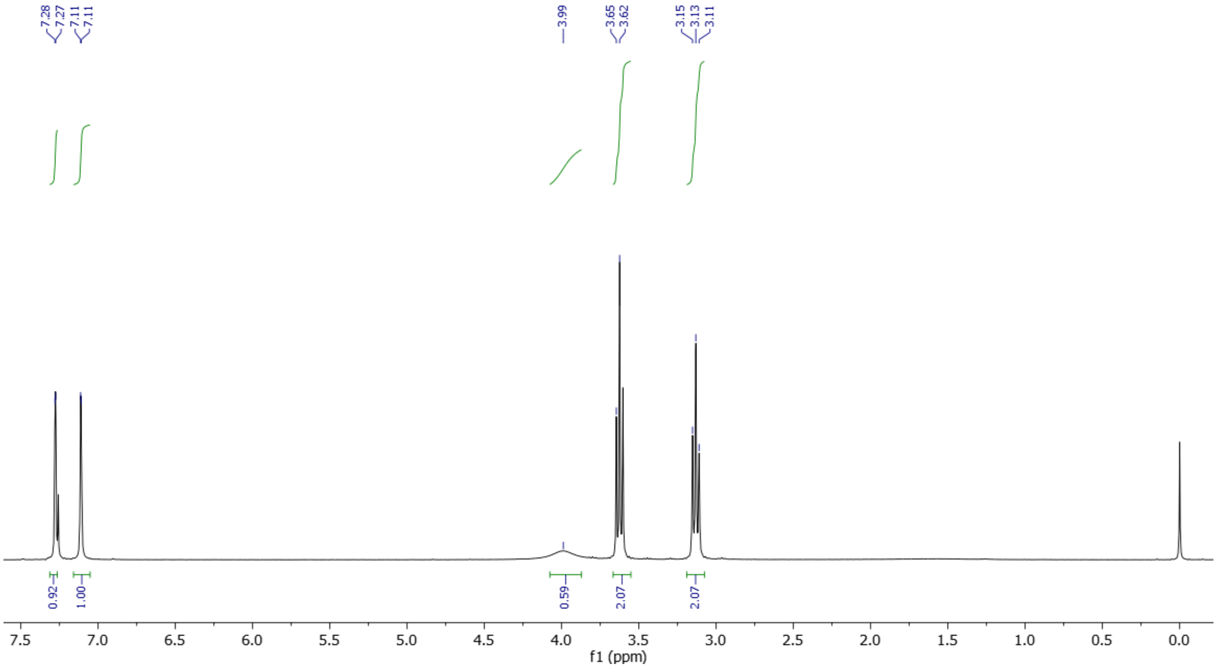

Figure S7. ^{1}H NMR (400 MHz, $CDCl_3$) spectrum of 5,7-dibromoindoline (5).

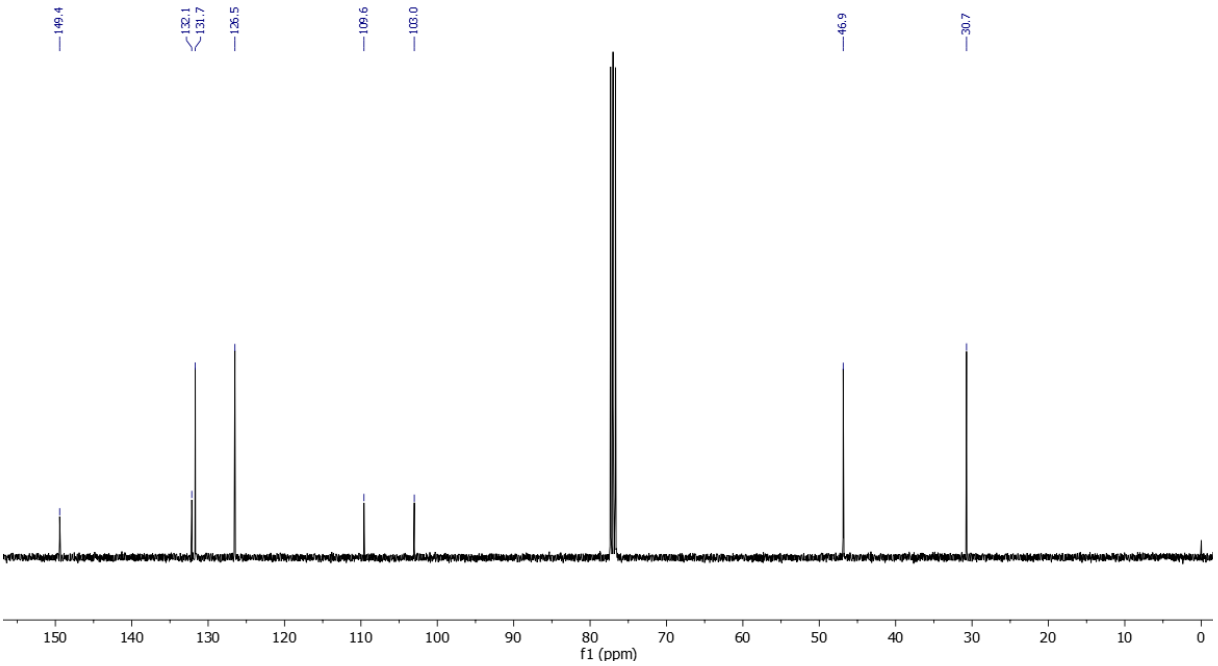

Figure S8. ^{13}C NMR (101 MHz, $CDCl_3$) spectrum of 5,7-dibromoindoline (**5**).

Synthesis of 5,7-dibromo-1-methoxy-1*H*-indole (4)

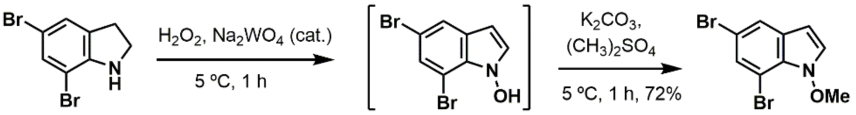

The following procedure is an adaptation from a previous report.[4] $Na_2WO_4{\cdot}2H_2O$ (0.36 g, 1.1 mmol) dissolved in water (5.0 mL) is added under stirring to a solution of 2,4-dibromoindoline **5** (1.5 g, 5.4 mmol) in MeOH/THF 3:1 (40 mL) at 5 °C. 30% H_2O_2 (6.1 mL, 54 mmol) is added over 5 min to the previous solution, and the resulting homogeneous mixture is vigorously stirred at 0 °C for 15 min and then at 10 °C for additional 30 min. The reaction turns from violet to light yellow. By combination of TLC and ESI-MS, the formation of the corresponding N-hydroxylated derivative (R*f* (toluene) = 0.29) is checked. Additional $Na_2WO_4{\cdot}2H_2O$ (0.283, 0.84 mmol) and 30% H_2O_2 (5 mL) are added, and the reaction is stirred at 10 °C for another 45 min. After ensuring complete consumption of the starting material, dimethyl sulfate (2.3 mL, 27 mmol) is added, followed by K_2CO_3 (3.0 g, 21.7 mmol) and the reaction is stirred for 1 h, checking the reaction progress by TLC. In case of any sign of precipitation, further THF (5-10 mL) is added, until the reaction mixture gets homogeneous

[4] K. Aoki, Y. Nagahama, K. Sugaya, Y. Maeda; H. Sato, K. Nakagawa(Goto), M. Somei, *Heterocycles* **2019**, *98*, 236–270

again. After this time, analysis by TLC shows no starting material and confirms the formation of the desired product R*f* (n-hex/DCM 2:1) = 0.54). Then, brine (200 mL) is added, and the crude is extracted with $CHCl_3$ (3×40 mL). The combined organic phases are washed with brine, dried over Na_2SO_4, and evaporated under reduced pressure to leave a yellowish syrup when cooled below 30 °C. This crude is purified by column chromatography (n-hex/DCM 5:1) to give 5,7-dibromo-1-methoxy-1*H*-indole **4** (1.2 g, 72%) as a white amorphous solid. R*f* (n-hex/DCM 2:1) = 0.54. ^{1}H NMR (400 MHz, $CDCl_3$): δ = 7.64 (d, *J* = 1.7 Hz, 1H, H-4), 7.51 (d, *J* = 1.7 Hz, 1H, H-6), 7.28 (d, *J* = 3.6 Hz, 1H, H-2), 6.31 (d, *J* = 3.5 Hz, 1H, H-3), 4.09 (s, 3H, C*H*$_3$). ^{13}C NMR (101 MHz, $CDCl_3$): δ = 131.7 (C-2), 130.9 and 130.7 (C-7a and C-3a), 128.9 (C-6), 125.5 (C-4), 115.6 (C-5), 105.2 (C-7), 100.8 (C-3), 70.4 (*C*H$_3$). ESI-MS: m/z = 325.4 $[M+Na]^+$; calcd. for $C_9H_7{}^{79}Br_2NNaO$: 325.9.

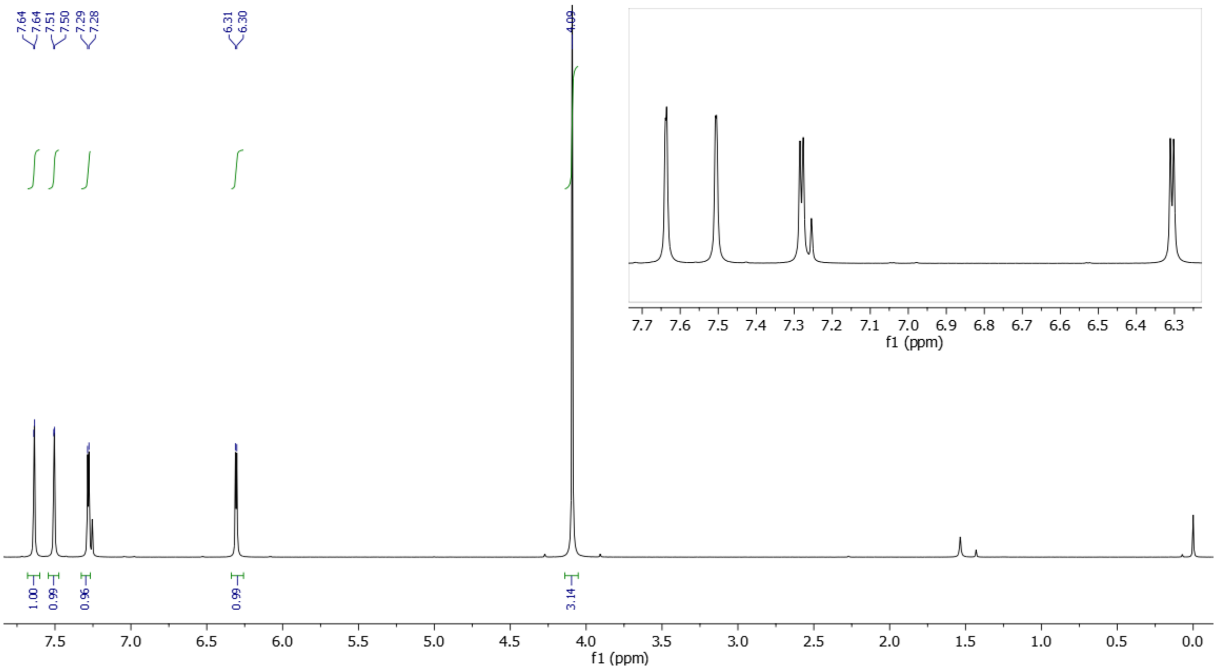

Figure S9. ^{1}H NMR (400 MHz, $CDCl_3$) spectrum of 5,7-dibromo-1-methoxy-1*H*-indole (**4**).

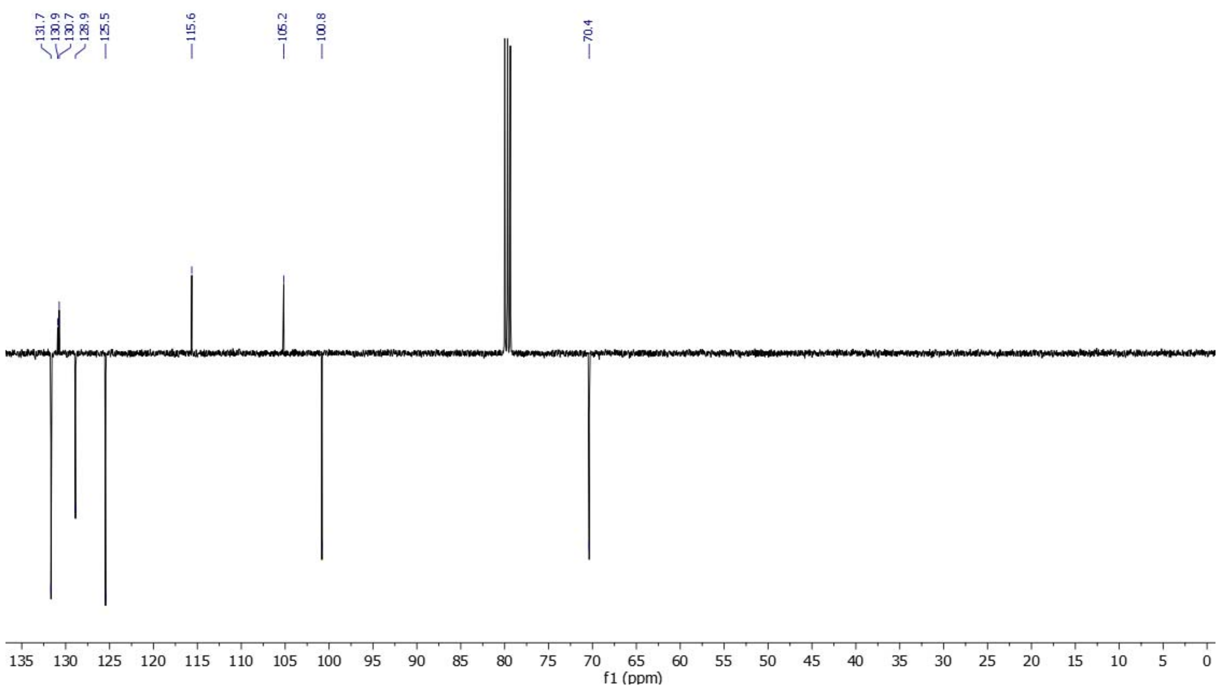

Figure S10. ^{13}C APT NMR (101 MHz, $CDCl_3$) spectrum of 5,7-dibromo-1-methoxy-1*H*-indole (**4**).

Synthesis of 5,7-dibromo-1-methoxy-1H-indole-3-carbonitrile (2)

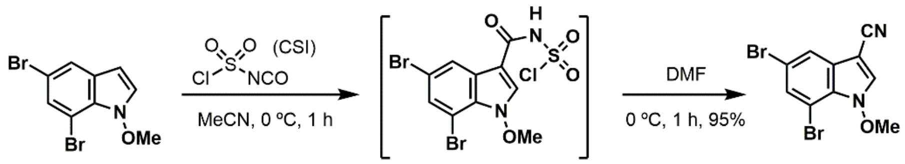

Following the reported procedure,[5] 5,7-dibromo-1-methoxy-1*H*-indole (1.0 g, 3. mmol) is dissolved in dry MeCN (20 mL). At 0 ºC, CSI (0.3 mL, 3.44 mmol) is added slowly. The reaction mixture is stirred for 1 h and maintained at 0ºC, after which the colorless precipitate formed is re-dissolved by DMF (10 mL). The solution is stirred for 1 h at 0 ºC, until TLC reveals quantitative transformation. The crude is poured into crushed ice, after which the resulting suspension is filtered and dried in a desiccator overnight. 5,7-dibromo-1-methoxy-1*H*-indole-3-carbonitrile **2**, (1.03 g, 95%) is obtained as a colorless solid, which is recrystallized from AcOEt. R*f* (n-hex/EtOAc 4:1) = 0.65. ^{1}H NMR (400 MHz, $CDCl_3$): δ = 7.84 (d, J = 1.7 Hz, 1H, C-4), 7.79 (s, 1H, C-2), 7.67 (d, J = 1.7 Hz, 1H, C-6), 4.18 (s, 3H, CH_3). ^{13}C NMR (101 MHz, $CDCl_3$): δ = 131.9 and 131.7 (C-2 and C-6), 127.7 and 127.4

[5] a) H. Vorbrüggen, K. Krolikiewicks, *Tetrahedron* **1994**, *50*, 6549–6558; b) S. Cascioferro, G. L. Petri, B. Parrino, D. Carbone, N. Funel, C. Bergonzini, G. Mantini, H. Dekker, D. Geerke, G. J. Peters, G. Cirrincione, E. Giovannetti, P. Diana, *Eur. J. Med. Chem.* **2020**, *189*, 112088..

(C-3a and C-7a), 121.8 (C-4), 116.2 (CN), 113.5 (C-5), 103.5 (C-7), 82.9 (C-3), 69.0 (CH_3). ESI-MS: m/z = 351.0 [M+Na]$^+$; calcd. for $C_{10}H_6{}^{79}Br_2N_2NaO$: 350.8.

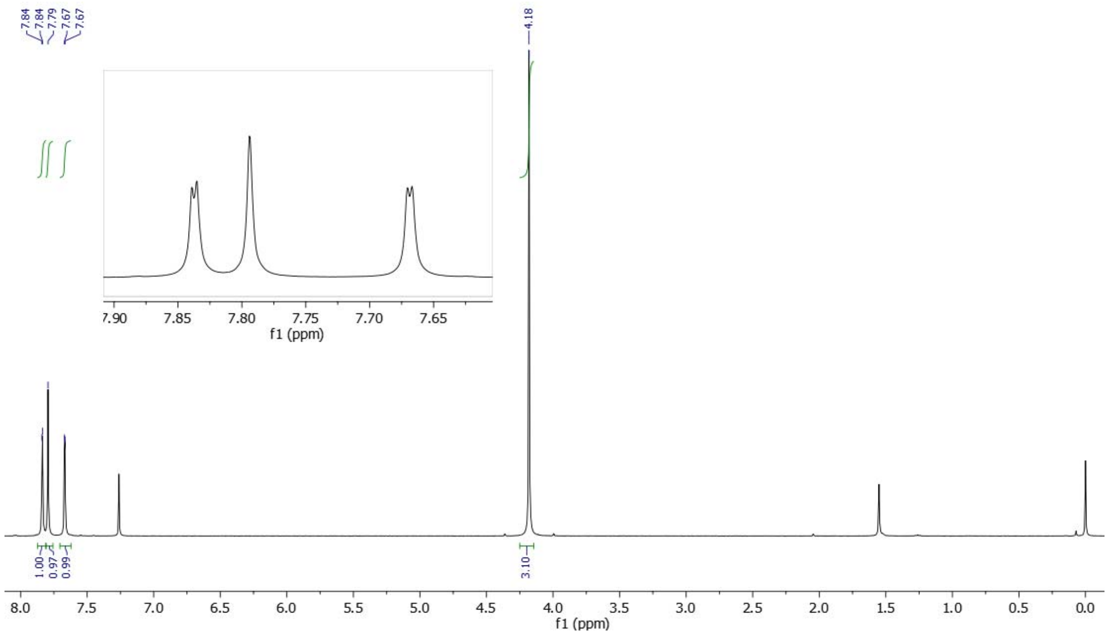

Figure S11. ^{1}H NMR (400 MHz, $CDCl_3$) spectrum of 5,7-dibromo-1-methoxy-1*H*-indole-3-carbonitrile (**2**).

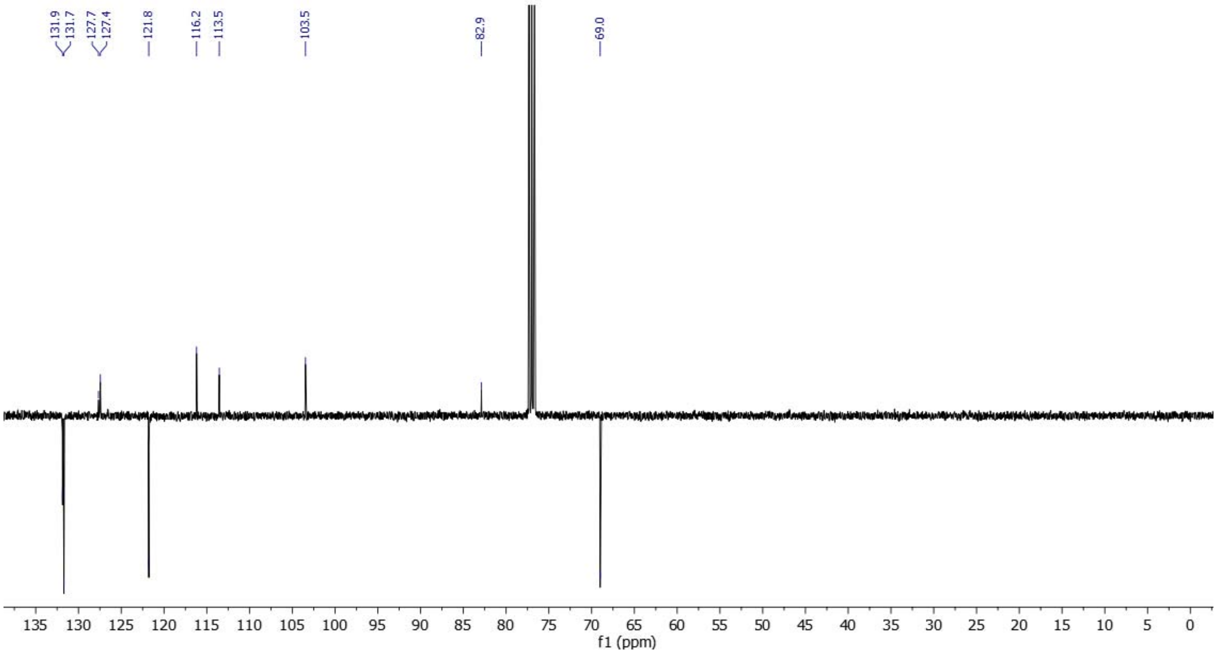

Figure S12. ^{13}C APT NMR (101 MHz, $CDCl_3$) spectrum of 5,7-dibromo-1-methoxy-1*H*-indole-3-carbonitrile (**2**).

Synthesis of 3,5-dibromo-1*H*-indole (3b)

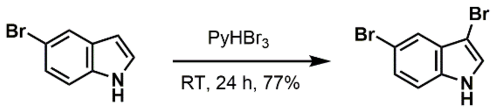

5-Bromoindole (0.4 g, 2.0 mmol) is mixed with $PyHBr_3$ (0.72 g, 2.2 mmol) in DCM (20 mL). At room temperature, the reaction is stirred overnight. The crude reaction mixture is diluted with chloroform (30 ml) and washed successively with sodium bisulfate (10%), sodium bicarbonate (sat. soln.), and brine. The organic phase is concentrated at reduced pressure and purified by column chromatography (n-hex/EtOAc 10:1) to afford the 3,5-dibromo-1*H*-indole **3b** (0.43 g, 77%) as a colorless solid. To avoid decomposition, the compound is stored at -20 ºC. R*f* (toluene) = 0.55. ^{1}H NMR (400 MHz, $CDCl_3$): δ = 8.23 (s, 1H), 7.72 (s, *J* = 1.8 Hz, 1H, H-4), 7.33 (dd, *J* = 8.7, 1.9 Hz, 1H, H-6), 7.24 (d, *J* = 8.7 Hz, 1H, H-7), 7.22 (s, 1H, H-2). ^{13}C NMR (101 MHz, $CDCl_3$): δ = 134.0 (C-7a), 128.6 (C-3a), 126.2 (C-6), 124.5 (C-2), 121.90 (C-4), 114.0 (C-5), 112.8 (C-7), 91.0 (C-3). ESI-MS: m/z = 271.7 $[M-H]^-$; calcd. for $C_8H_4{}^{79}Br_2N$: 271.9.

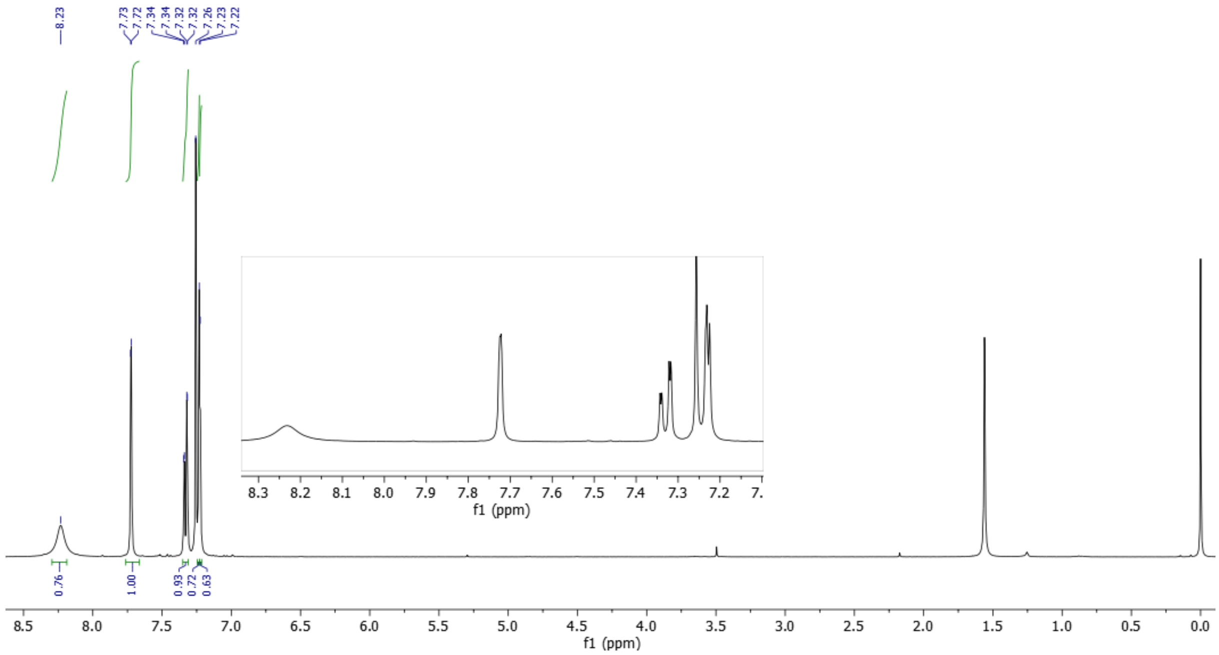

Figure S13. ^{1}H NMR (400 MHz, $CDCl_3$) spectrum of 3,5-dibromo-1*H*-indole (**3b**).

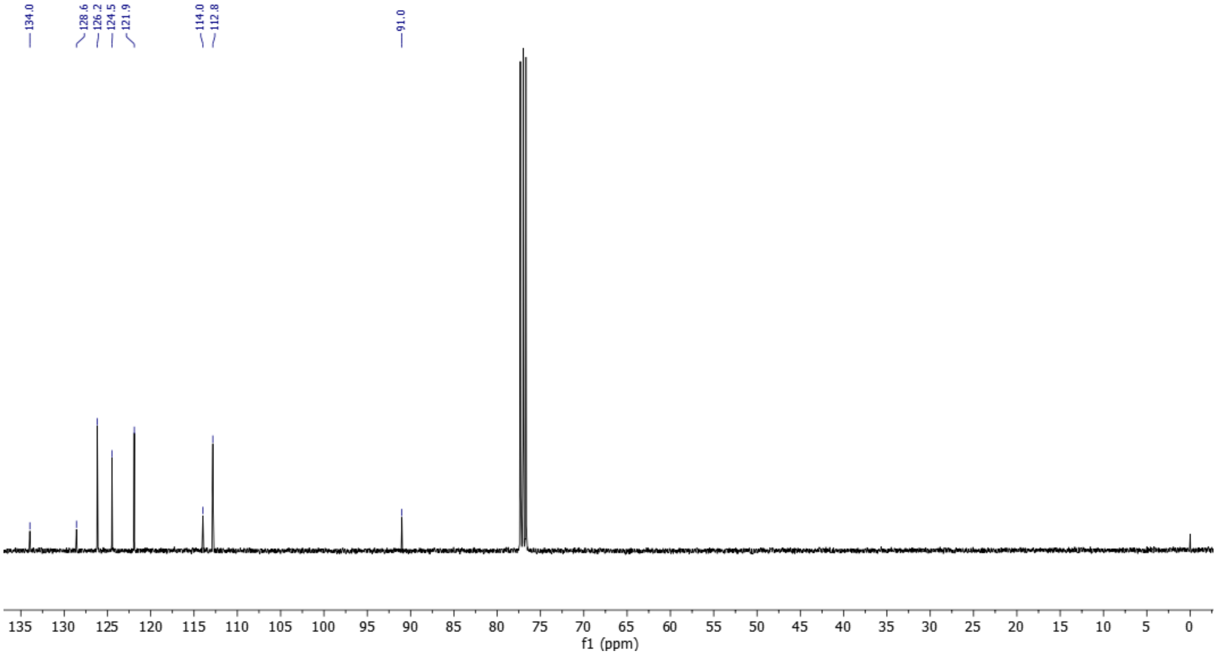

Figure S14. ^{13}C NMR (101 MHz, $CDCl_3$) spectrum of 3,5-dibromo-1*H*-indole (**3b**).

2. Synthesis of 2,3,5-tribromo-1*H*-indole (3c)

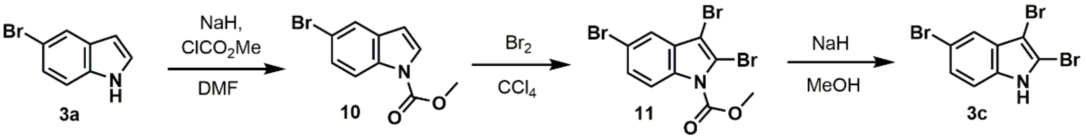

Synthesis of methyl 2,3,5-tribromo-1*H*-indole-1-carboxylate (11)

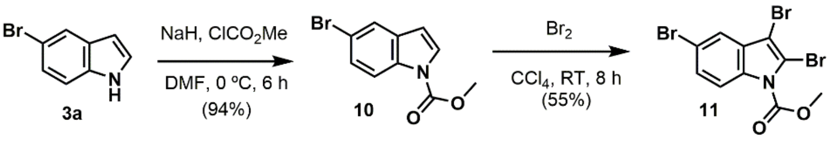

5-Bromoindole **3a** (1 g, 5.1 mmol) is mixed with 60% NaH in mineral oil (0.24 g, 6.1 mmol) in DMF at 0 ºC. After 20 min of stirring, methyl chloroformate (0.47 mL, 6.1 mmol) is added, and the reaction is stirred until total consumption of the starting material. The reaction is quenched with sat. soln. of NH_4Cl, diluted with AcOEt (100 mL), washed with brine, and dried over Na_2SO_4. After removing the solvent by evaporation at reduced pressure, the methyl 5-bromo-1*H*-indole-1-carboxylate **10** (1.22 g, 94%) is obtained sufficiently pure and, therefore, used in the next step without further purification. Selective 2,3-dibromination was performed according to a previous report.[6] In a 250 mL round bottom flask, **10** (0.4 g, 1.6 mmol) is dissolved in CCl_4 (80 mL). Bromine (0.64 mL, 12.6 mmol), previously dissolved in CCl_4 (5 mL), is added over 10 min. The reaction is stirred at room temperature for 12 h. The resulting solution is quenched with sat. soln. of $Na_2S_2O_3$, washed with sat. soln. of $NaHCO_3$, brine, and dried over Na_2SO_4. After removing the solvent by evaporation at reduced pressure, the crude is purified by column chromatography (n-hex/toluene 1:1) to afford methyl 2,3,5-tribromo-1*H*-indole-1-carboxylate **11** (0.36 g, 55%) as colorless solid. R*f* (n-hex/toluene 1:2) = 0.52. ^{1}H NMR (400 MHz, $CDCl_3$): δ = 7.95 (d, *J* = 8.8 Hz, 1H, H-7), 7.64 (d, *J* = 2.0 Hz, 1H (H-4)), 7.44 (dd, *J* = 9.0, 2.0 Hz, 1H, H-6), 4.09 (s, 3H, C*H*$_3$). ^{13}C NMR (101 MHz, $CDCl_3$): δ = 150.3 (*C*O$_2$Me), 134.4 (C-7a), 130.0 (C-3a), 128.2 (C-3), 121.9 (C-4), 117.6 (C-5), 117.0 (C-7), 112.5 (C-2), 104.3 (C-3), 54.3 (*C*H$_3$). ESI-MS: m/z = 431.7 $[M+Na]^+$; calcd. for $C_{10}H_6{}^{79}Br_3NNaO_2$: 431.8.

[6] G. Chelucci, G. A. Pinna, G. Pinna, *European J. Org. Chem.* **2014**, *2014*, 3802–3807.

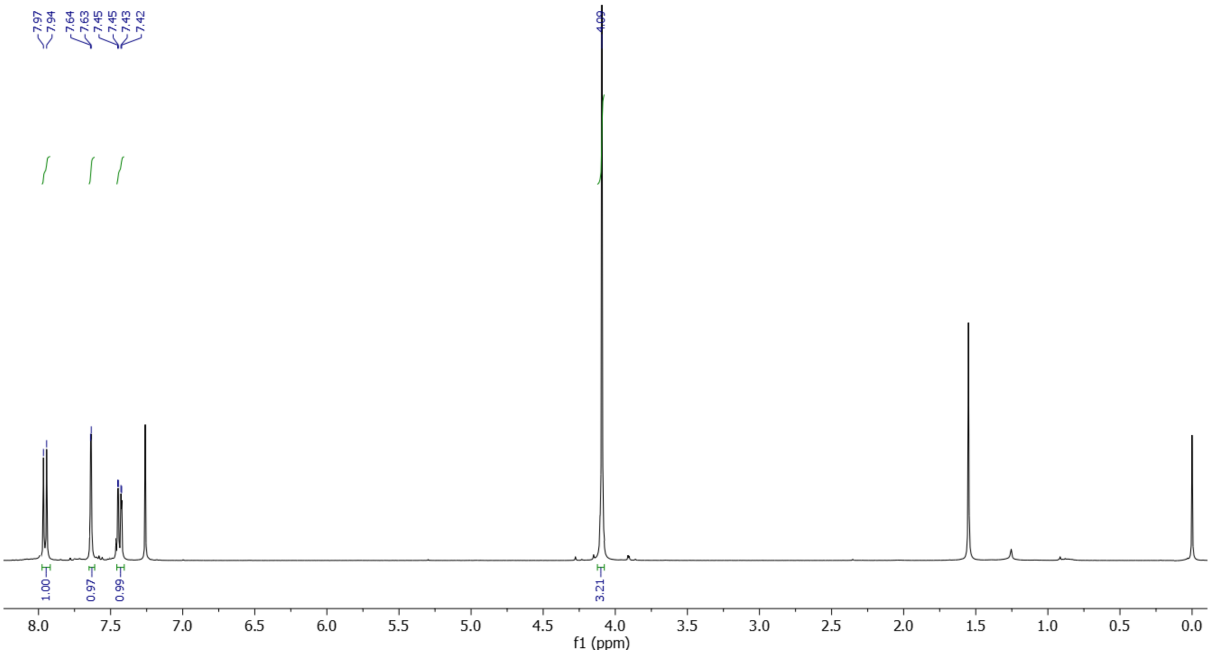

Figure S15. ^{1}H NMR (400 MHz, $CDCl_3$) spectrum of methyl 2,3,5-tribromo-1*H*-indole-1-carboxylate (**11**).

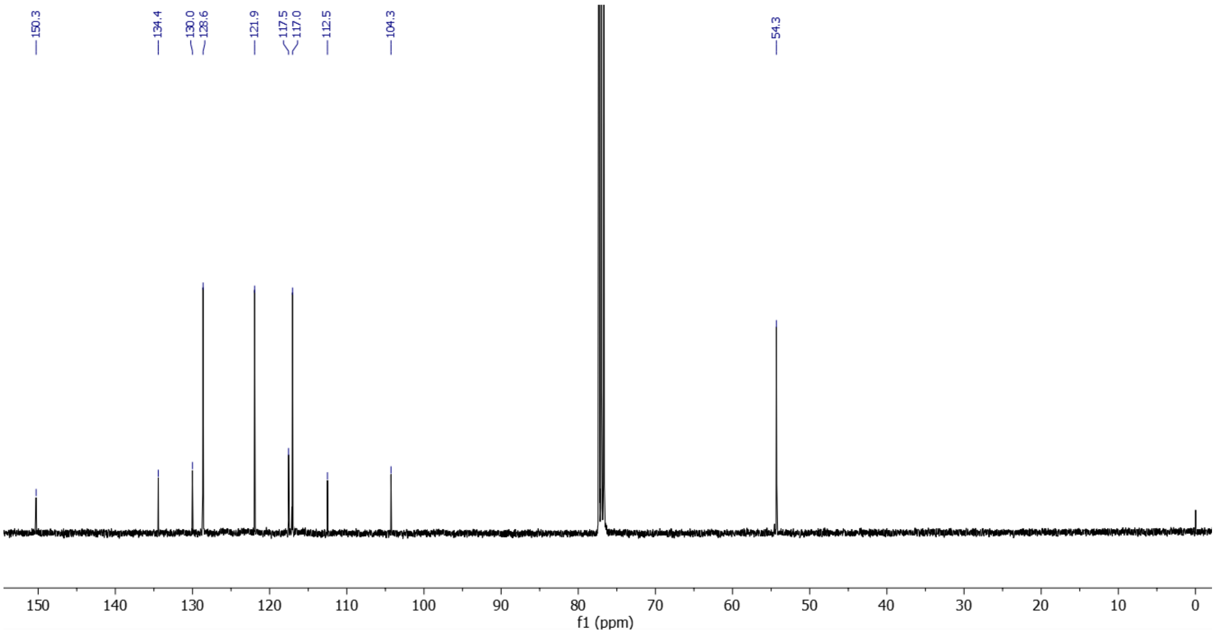

Figure S16. ^{13}C NMR (101 MHz, $CDCl_3$) spectrum of methyl 2,3,5-tribromo-1*H*-indole-1-carboxylate (**11**).

Synthesis of 2,3,5-tribromo-1*H*-indole (3c)

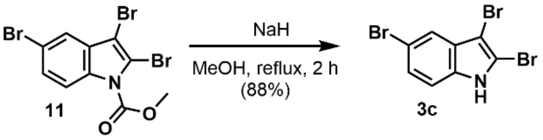

Methyl 2,3,5-tribromo-1*H*-indole-1-carboxylate **11** (0.3 g, 0.73 mmol) is suspended in MeOH (5 mL). 60% NaH in mineral oil (44 mg, 1.1 mmol) is carefully added. The reaction mixture is refluxed for 2 h and then quenched with sat. soln. of NH_4Cl, diluted with AcOEt (40 mL), washed with brine, and dried over Na_2SO_4. After removing the solvent by evaporation at reduced pressure, the resulting crude product is purified by column chromatography (n-hex/AcOEt 10:1) to afford the 2,3,5-tribromo-1*H*-indole **3c** (0.23 g, 88%) pure as a white solid. R*f* (n-hex/AcOEt 4:1) = 0.58. ^{1}H NMR (400 MHz, $CDCl_3$): δ = 8.30 (s, 1H, NH), 7.64 (d, *J* = 1.9 Hz, 1H, H-4), 7.31 (dd, *J* = 8.7, 1.9 Hz, 1H, H-6), 7.17 (d, *J* = 8.6 Hz, 1H, H-7). ^{13}C NMR (101 MHz, $CDCl_3$): δ = 134.2 (C-7a), 129.1 (C-3a), 126.5 (C-6), 121.5 (C-4), 114.6 (C-5), 112.1 (C-7), 111.2 (C-2), 93.8 (C-3). ESI-MS: m/z = 349.6 $[M-H]^-$; calcd. for $C_8H_3{}^{79}Br_3N$: 349.8.

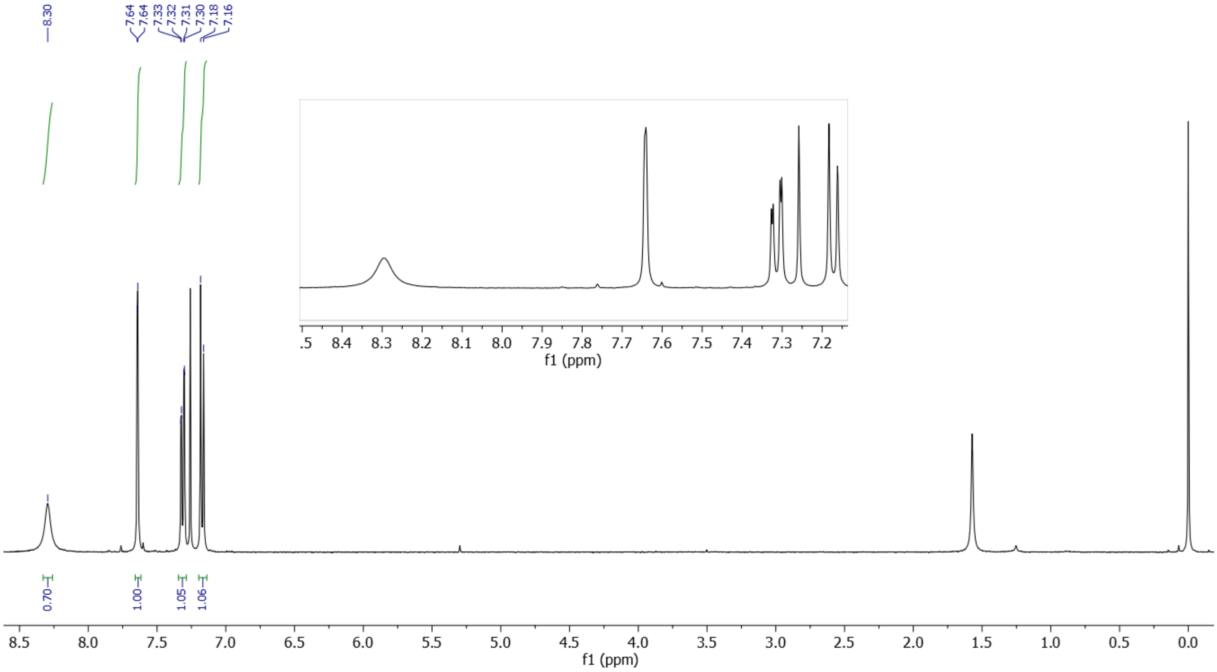

Figure S17. ^{1}H NMR (400 MHz, $CDCl_3$) spectrum of 2,3,5-tribromo-1*H*-indole (**3c**).

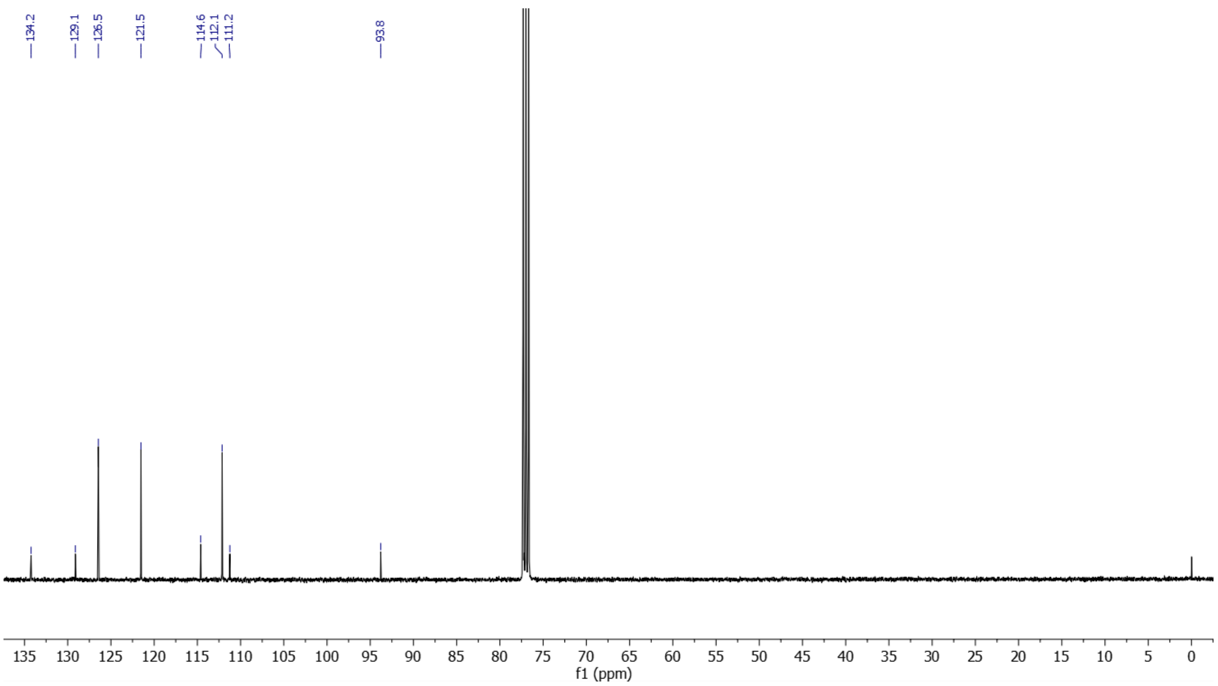

Figure S18. ^{13}C NMR (101 MHz, $CDCl_3$) spectrum of 2,3,5-tribromo-1*H*-indole (**3c**).

Synthesis and characterization of biindoles

General procedure for Somei-Michael reaction

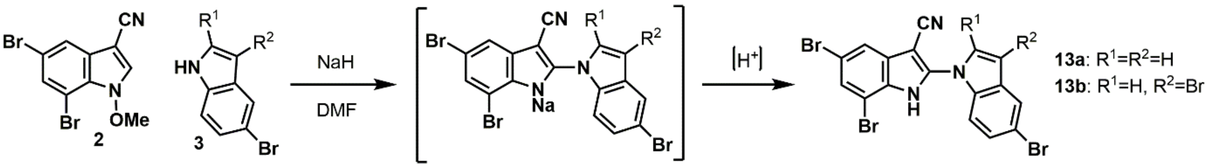

Indole derivative **3** (0.3 mmol) is dissolved in dry DMF (5 mL) and the solution is cooled at 0 °C. NaH (0.33 mmol, 1.1 equiv) is added in portions over 10 min and the mixture is stirred for 1 h. Maintaining the temperature at 0 °C, indole derivative **2** (0.36 mmol, 1.2 equiv), previously dissolved in DMF (2 mL) is slowly added and the reaction mixture is stirred for 12 h at room temperature. The reaction is quenched with sat. soln. of NH_4Cl, diluted with AcOEt (100 mL), washed with brine, and dried over Na_2SO_4. After removing the solvent by evaporation at reduced pressure, the crude mixture obtained is triturated with MeOH to dissolve excess of starting material and byproducts to afford the insoluble biindole ~95% pure as a colorless solid in 50-70% yield.

Synthesis of biindole 13a

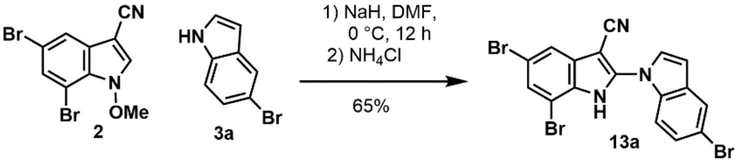

Following the general procedure for the Somei coupling described above, indole **2** (100 mg, 0.30 mmol) and **3a** (71 mg, 0.36 mmol) are combined with 60% NaH in mineral oil (13.3 mg, 0.33 mmol) in DMF (5 ml) to afford the biindole **13a** (97 mg, 65%) as a colorless solid. R*f* (toluene/n-hex 2:1) = 0.32. M.p. = 288-290 ºC. ^{1}H NMR (400 MHz, THF-d_8): δ = 12.19 (s, 1H, NH), 7.91 (d, J = 1.7 Hz, 1H, H-4’), 7.85 (d, J = 2.0 Hz, 1H, H-4), 7.72 (d, J = 1.7 Hz, 1H, H-6’), 7.68 (d, J = 3.5 Hz, 1H, H-2), 7.53 (d, J = 8.8 Hz, 1H, H-7), 7.42 (dd, J = 8.8, 2.0 Hz, 1H, H-6), 6.80 (d, J = 3.5, 1H, H-3). ^{13}C NMR (101 MHz, THF-d_8): δ = 142.4 (C-2’), 135.9 (C-7a), 132.4 (C-3a’), 132.1 (C-3a), 130.4 (C-7a’), 130.3 (C-2), 130.1 (C-6’), 127.0 (C-6), 124.6 (C-4’), 121.8 (C-4), 116.3 (C-5), 115.9 (C-5’), 113.7 (C-7), 113.3 (CN), 106.9 (C-7’), 106.5 (C-3), 82.2 (C-3’). HR-MS: m/z = 489.8207 [M-H]$^-$; calcd. for $C_{17}H_7N_3{}^{79}Br_3$: 489.8196.

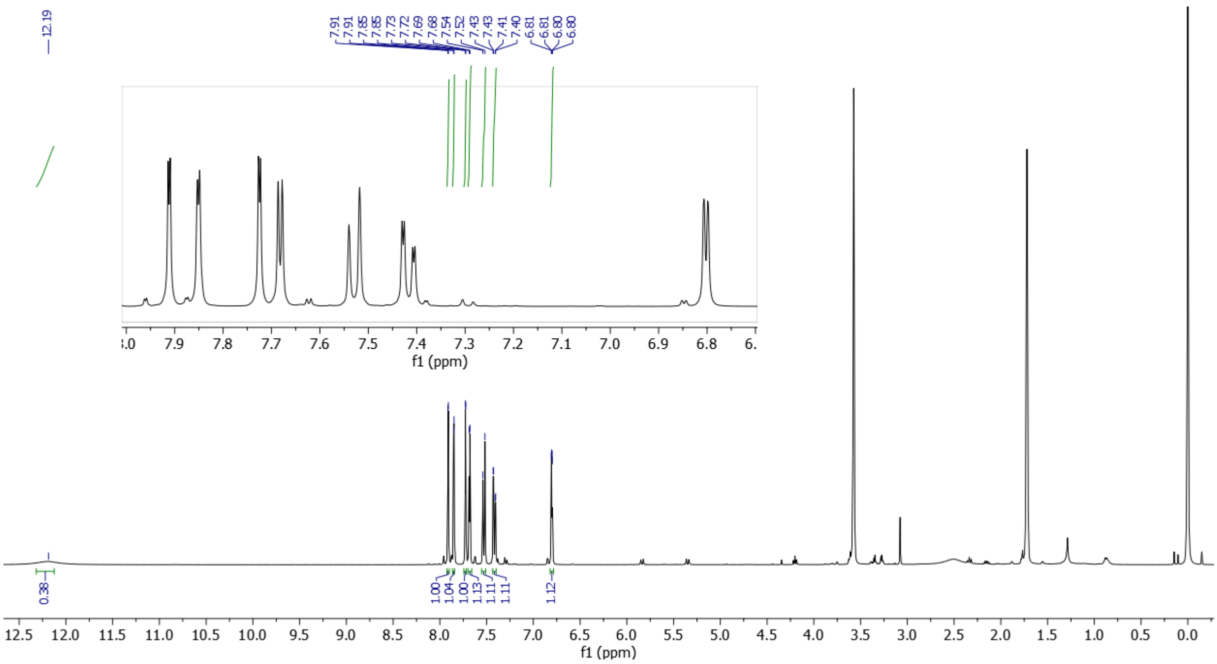

Figure S19. ^{1}H NMR (400 MHz, THF-d_8) spectrum of biindole **13a**.

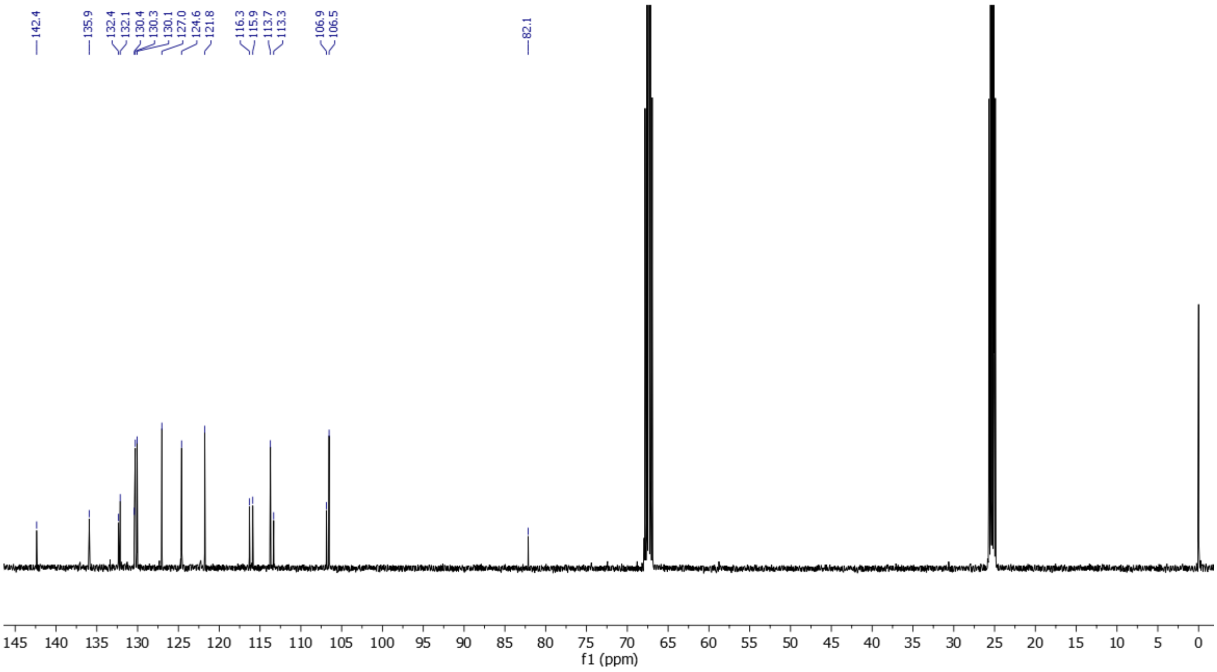

Figure S20. ^{13}C NMR (101 MHz, THF-d_8) spectrum of biindole **13a**.

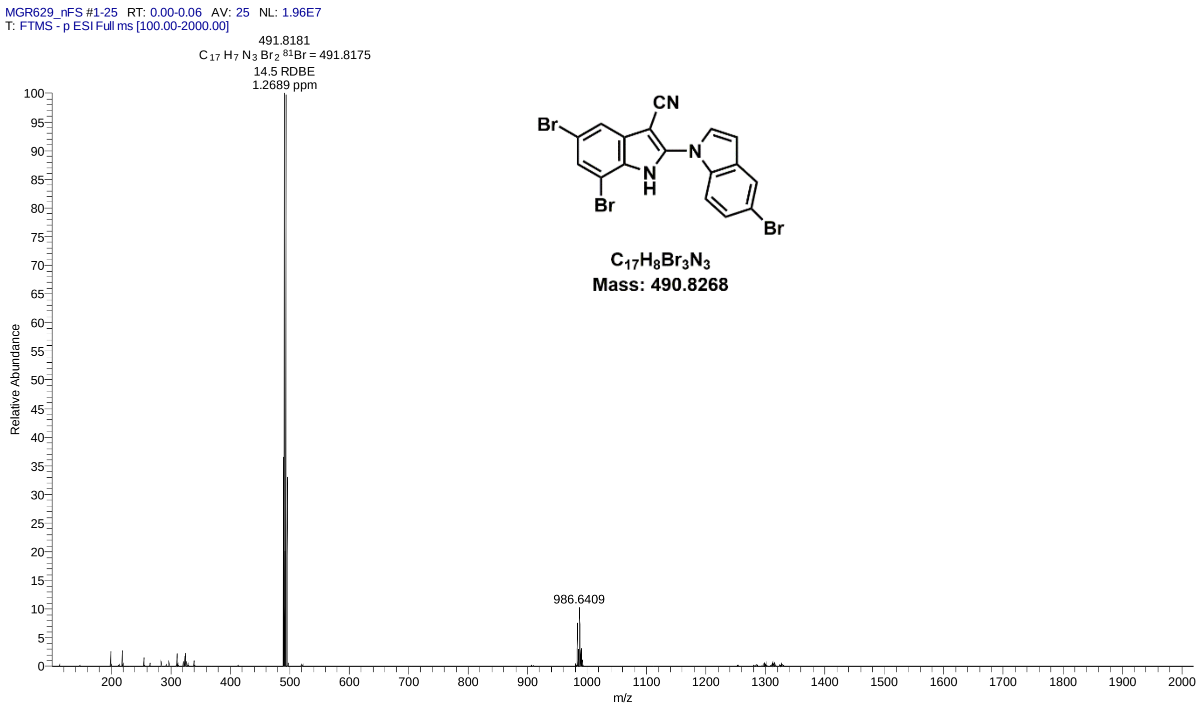

Figure S21. HR-MS (negative mode) of biindole **13a**.

Synthesis of the biindole 13b

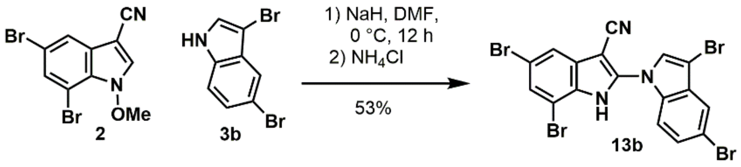

Following the general procedure for the Somei coupling described above, indole **2** (100 mg, 0.30 mmol) and **3b** (83 mg, 0.36 mmol) are combined with 60% NaH in mineral oil (13.3 mg, 0.33 mmol) in DMF (5 ml) to afford the biindole **13b** (92 mg, 53%) as a colorless solid.. R*f* (toluene/n-hex 2:1) = 0.36. M.p. = 290-292 ºC. ^{1}H NMR (400 MHz, THF-d_8): δ = 12.35 (s, 1H, NH), 7.95 (d, *J* = 1.6 Hz, 1H, H-4'), 7.92 (s, 1H, H-2), 7.78 (d, *J* = 1.8 Hz, 1H, H-4), 7.76 (d, *J* = 1.6 Hz, 1H, H-6'), 7.57 (d, *J* = 8.7 Hz, 1H, H-7), 7.52 (dd, *J* = 8.7, 1.8 Hz, 1H, H-6). ^{13}C NMR (101 MHz, THF-d_8): δ = 141.1 (C-2'), 138.1 (C-7a), 135.5 (C-3a'), 132.4 (C-3a), 130.3 (C-7a'), 129.6 (C-6'), 128.5 (C-6), 125.9 (C-2), 122.9 (C-4'), 121.9 (C-4), 116.8 (C-5), 116.5 (C-5'), 114.3 (C-7), 113.1 (CN), 106.9 (C-7'), 95.4 (C-3), 82.7 (C-3'). HR-MS: m/z = 567.7313 [M-H]$^{-}$; calcd. for $C_{17}H_6N_3{}^{79}Br_4$: 567.7301.

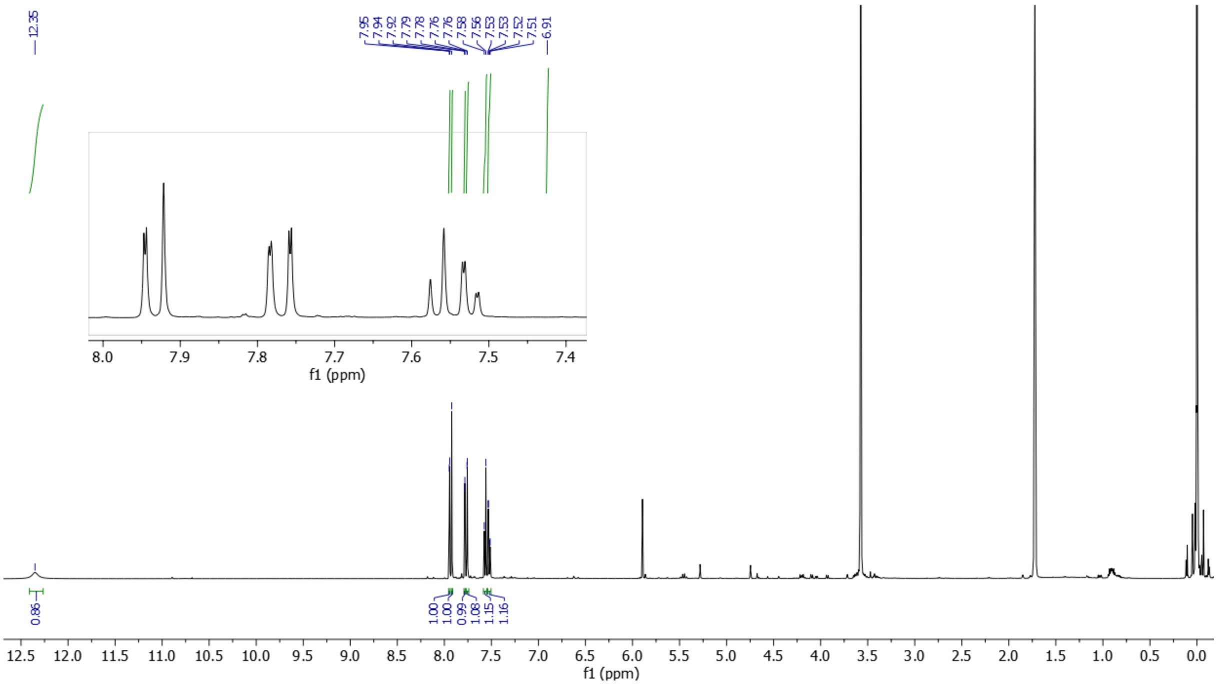

Figure S22. ^{1}H NMR (400 MHz, $CDCl_3$) spectrum of biindole **13b**.

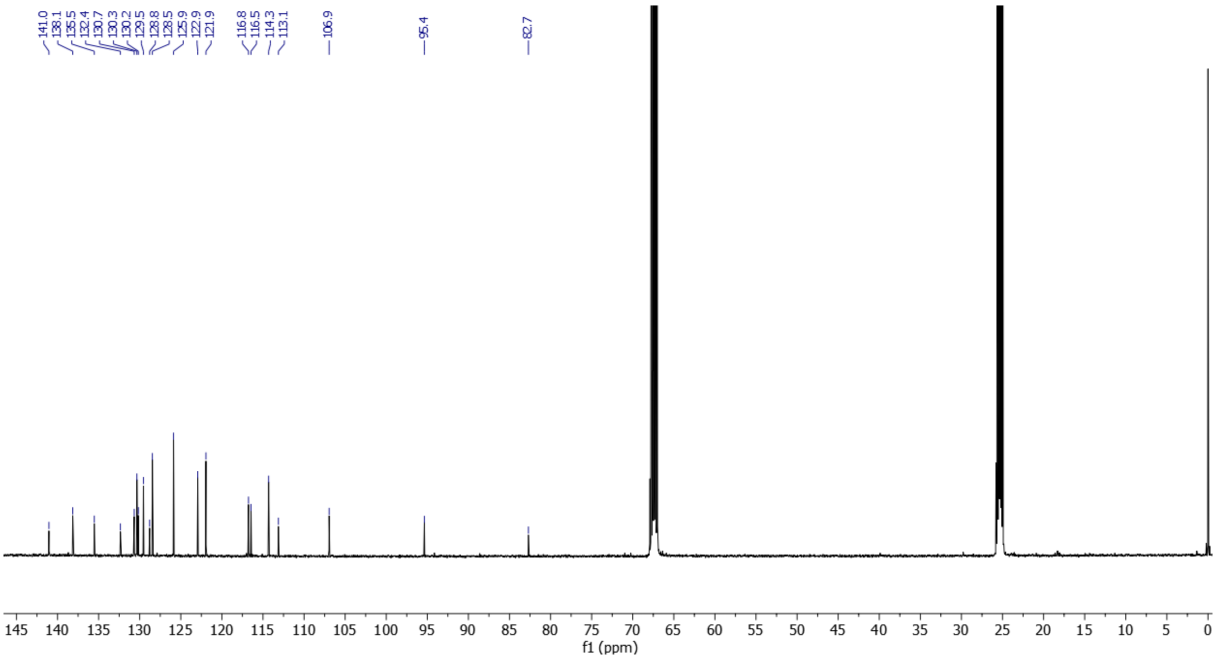

Figure S23. ^{13}C NMR (101 MHz, $CDCl_3$) spectrum of biindole **13b**.

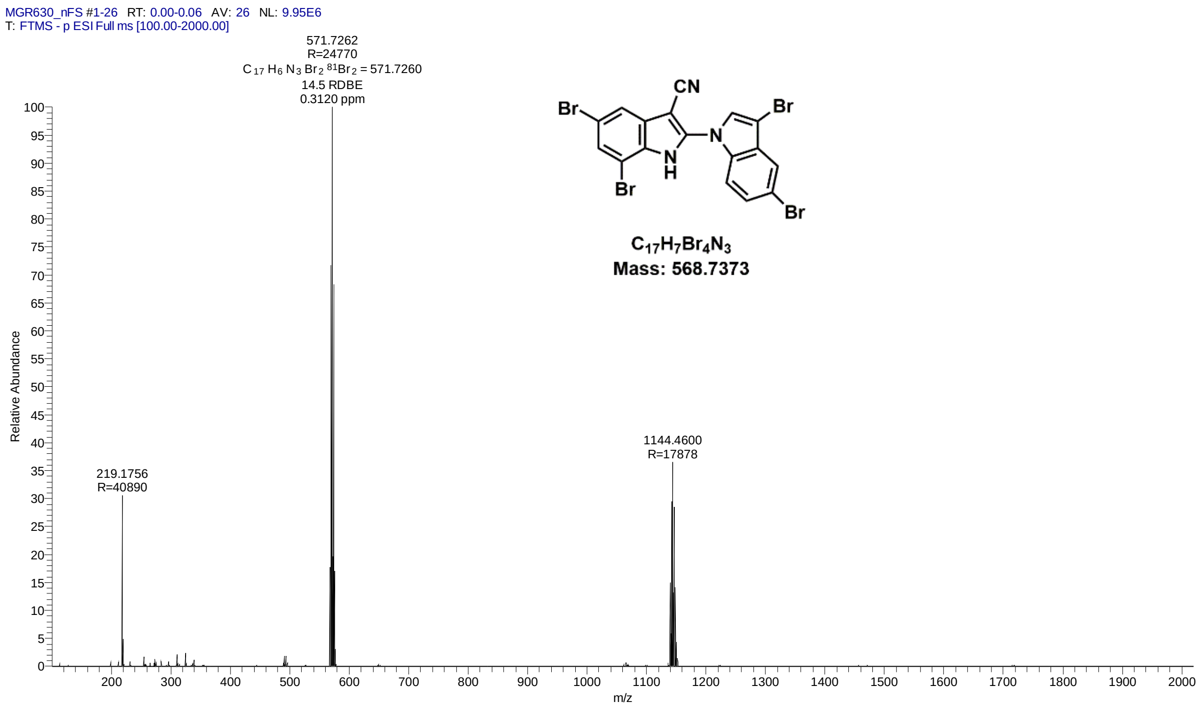

Figure S24. HR-MS (negative mode) of biindole **13b**.

Failed attempts to synthesize AETX (1) by direct coupling of 2 and 3c

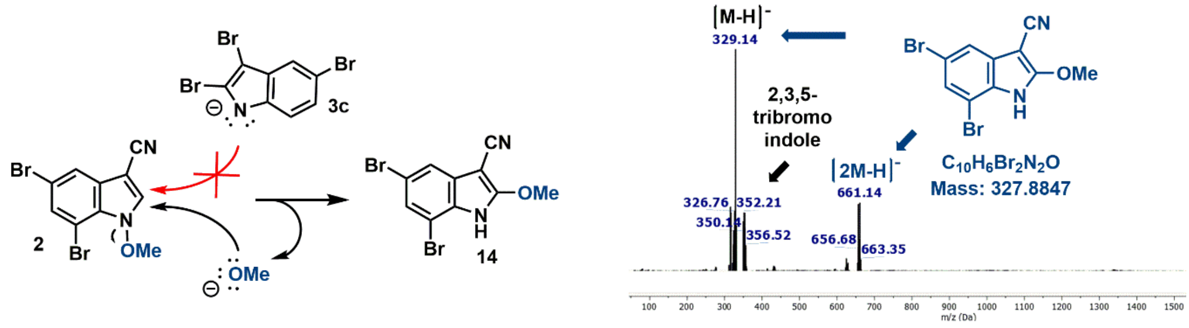

Following the general procedure for the Somei coupling described above, indole **2** (100 mg, 0.30 mmol) and **3c** (83 mg, 0.36 mmol) are combined with 60% NaH in mineral oil (13.3 mg, 0.33 mmol) in DMF (5 ml). Unfortunately, the expected biindole was not obtained despite total consumption of the starting material **2**. Instead, ESI-MS analysis of the reaction crude reveals the formation of the indole species **14**. This byproduct corresponds to the auto decomposition of **2**, when the methoxide anion, initially generated by a reductive cleavage of N-OMe with NaH, is the nucleophile engaging in the Michael addition. This side reaction is

described in detail by Somei *et al.* when poor nucleophiles are used.[7] Changing the reaction conditions was unsuccessful. Thus, the same outcome is obtained even when; 1) **3c** is used in higher amounts, 2) other bases (e.g., KH, K*t*-BuO or Cs_2CO_3) are used, 3) different catalyst with success in Michael-Type reactions are applied while using indole as nucleophile (i.e., Cu^I in combination with phosphine or $[imid]^+Cl^-$ ligands[8]).

Synthesis of biindole 16

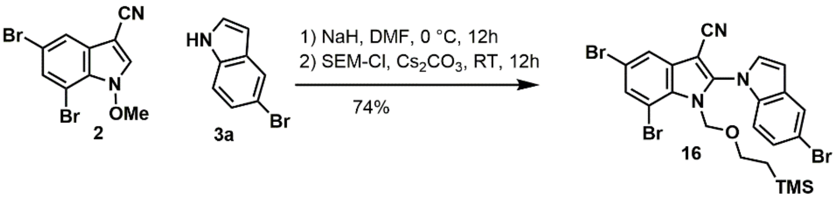

Indoles **2** (200 mg, 0.60 mmol) and **3a** (142 mg, 0.72 mmol) are combined with 60% NaH in mineral oil (26.6 mg, 0.66 mmol) in DMF (10 ml) according to the general procedure described above. However, instead of quenching the reaction with NH_4Cl, SEM-Cl (0.26 mL, 1.5 mmol) was added, followed by Cs_2CO_3 (0.39 g, 1.2 mmol), and the reaction is stirred for additional 12 h at room temperature. Then, the reaction is quenched with sat. soln. of NH_4Cl, diluted with AcOEt (100 mL), washed with brine, and dried over Na_2SO_4. After removing the solvent by evaporation at reduced pressure, the crude is purified by column chromatography (n-hex/toluene 3:1) to afford biindole **16** (280 mg, 74%) as a colorless solid. R*f* (toluene/n-hex 1:1) = 0.32. ^{1}H NMR (400 MHz, $CDCl_3$): δ = 7.89 (d, *J* = 1.7 Hz, 1H, H-4'), 7.86 (d, *J* = 1.8 Hz, 1H, H-4), 7.77 (d, *J* = 1.7 Hz, 1H, H-6'), 7.41 (dd, *J* = 8.7, 1.9 Hz, 1H, H-6), 7.36 (d, *J* = 3.4 Hz, 1H, H-2), 7.15 (d, *J* = 8.7 Hz, 1H, H-7), 6.81 (d, *J* = 3.4, 1H, H-3), 5.74 (d, *J* = 10.5 Hz, 1H, N-C*H*$_2$-O), 5.25 (d, *J* = 10.5 Hz, 1H, N-C*H*$_2$-O), 3.23 – 3.15 (m, 2H, O-C*H*$_2$), 0.77 – 0.70 (m, 2H, C*H*$_2$-Si), -0.11 (s, 9H, Si(C*H*$_3$)$_3$). ^{13}C NMR (101 MHz, $CDCl_3$): δ = 142.1 (C-2'), 135.6 (C-7a), 132.9 (C-2), 130.6 (C-3a'), 130.1 (C-3a), 130.1 (C-7a'), 129.3 (C-6'), 127.1 (C-6), 124.3 (C-4'), 121.7 (C-4), 116.9 (C-5), 115.7 (C-5'), 112.3 (CN), 112.0 (C-7), 106.6 (C-3), 106.3 (C-7'), 84.6 (C-3'), 72.8 (N-*C*H$_2$-O), 66.4 (O-*C*H$_2$), 17.7 (*C*H$_2$-Si), -1.6 (Si(*C*H$_3$)$_3$). ESI-MS: m/z = 643.8 $[M+Na]^+$; calcd. for $C_{23}H_{22}{}^{79}Br_3N_3NaOSi$: 643.9.

---

7 F. Yamada, D. Shinmyo, M. Nakajou, M. Somei, *Heterocycles* **2012**, *86*, 435–453.
8 S. Kim, S. Kang, G. Kim, Y. Lee, *J. Org. Chem.* **2016**, *81*, 4048–4057.

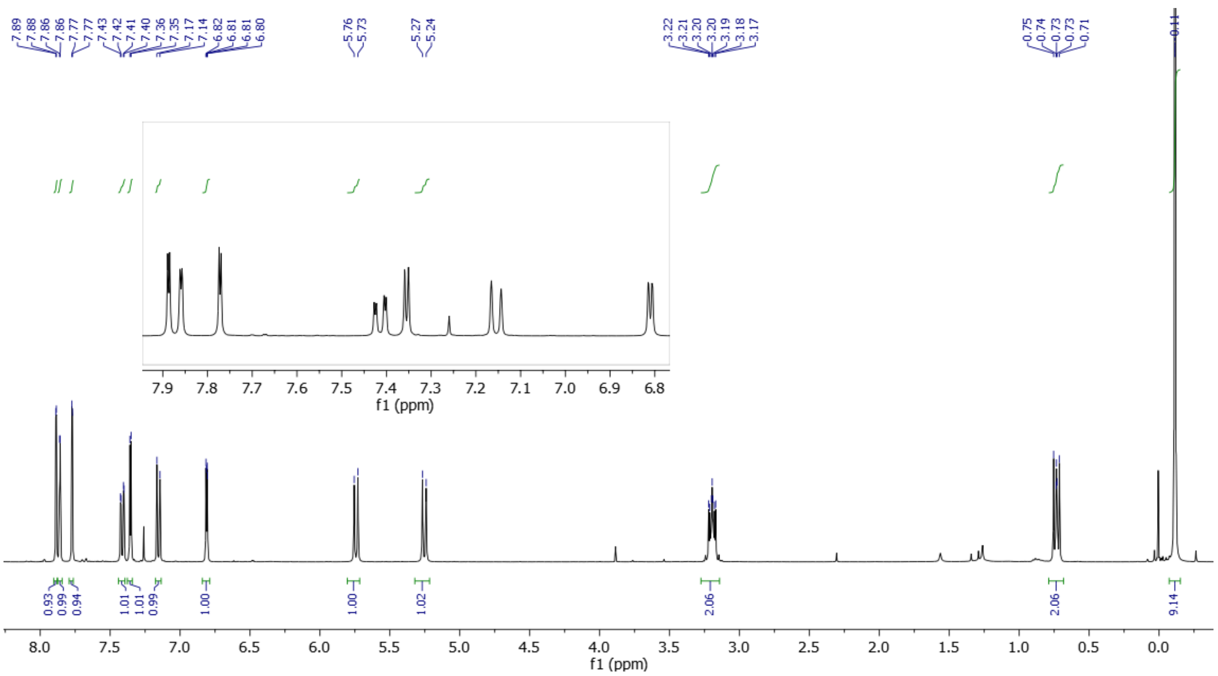

Figure S25. ^{1}H NMR (400 MHz, $CDCl_3$) spectrum of biindole **16**.

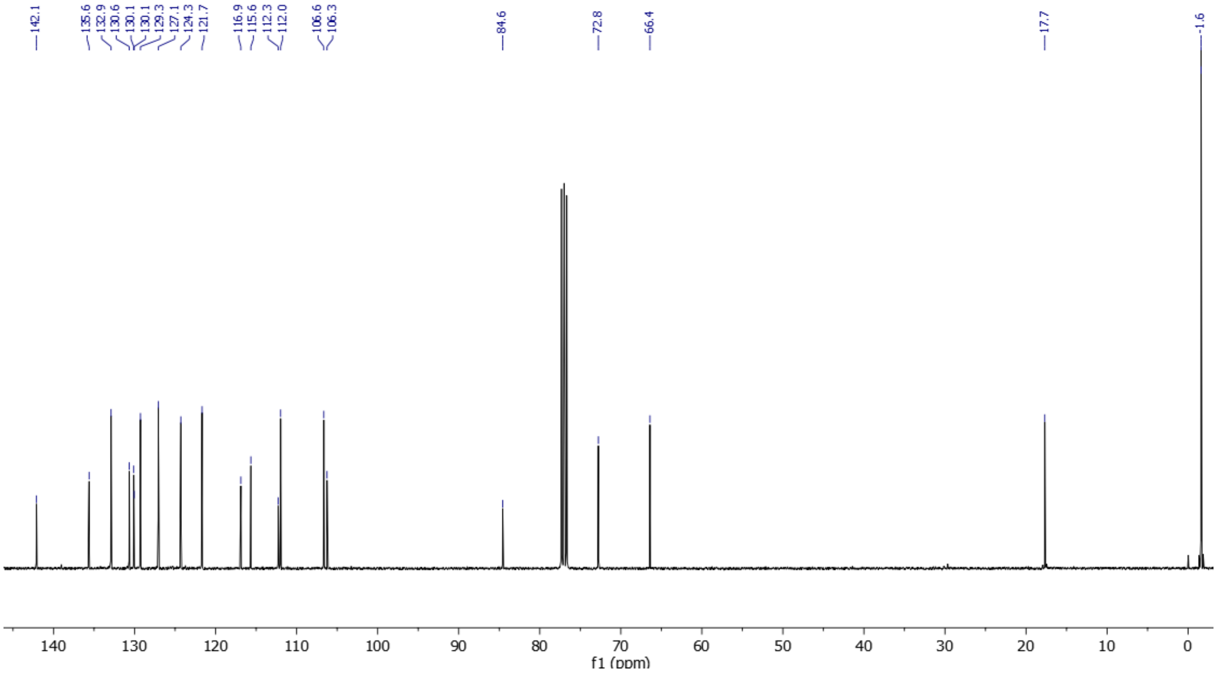

Figure S20. ^{13}C NMR (101 MHz, $CDCl_3$) spectrum of biindole **16**.

Bromination of biindoles

1. Bromination studies of 13a and 13b

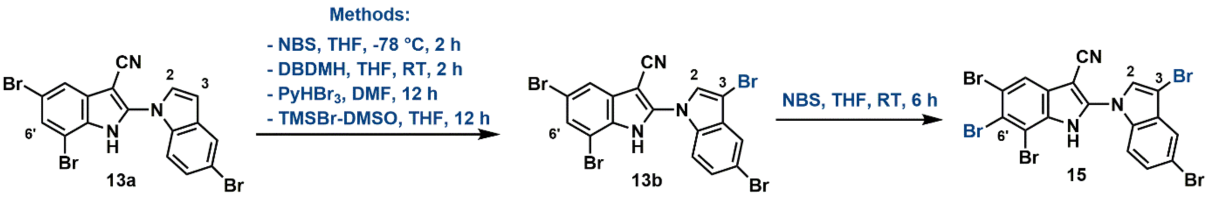

Attempts to brominate simultaneously C-2 and C-3 in **13a** or only C-2 in **13b** were performed using different brominating agents in THF or DMF as solvents. The insolubility of these biindoles in nonpolar solvents such as CCl_4, $CHCl_3$, DCM, and DCE considerably limited the bromination studies. Test reactions (20 mg scale) were performed using protocols with the following reagents: NBS,[9] DBDMH,[10] $PyHBr_3$,[11] and TMSBr-DMSO[12]. The progress of the reactions and, therefore, the consequent incorporation of bromine atoms was analyzed by ESI-MS in negative mode, due to the facile ionization of the NH group. As a result, it was concluded, that the mild brominating agent $PyHBr_3$ (3 equiv) in DMF efficiently transforms **13a** to **13b**. However, no further bromine atom is incorporated even when running the reaction for more than 24 h. Unfortunately, similar outcomes were observed when using TBSBr-DMSO (3 equiv each) in THF at room temperature. The use of DMBDH (3 equiv) in THF at room temperature also permits the formation of **13b**, although additional side reactions beside bromination arise when leaving the reaction to run for a longer time. Experiments using NBS are detailed as follows:

Bromination using NBS in THF

The method based on using NBS in THF at -78 ºC was initially considered the most promising attempt, based on the success in C-2,3 simultaneous dibromination of indole species achieved by Langer *et al.*[8] Moreover, the same procedure was used by Mathey *et al.* for the dibromination of an also sterically hindered *N*-Aryl-Indole in 89% yield.[13] Accordingly, **13a** (20 mg, 40 µmol) was treated with NBS (22 mg, 120 µmol, 3 equiv) reproducing the conditions reported. ESI-MS analysis after 1 h, 2 h, 3 h, and 4 h of reaction

[9] M. Hussain, S. M. Tengho Toguem, R. Ahmad, D. Thanh Tùng, I. Knepper, A. Villinger, P. Langer, *Tetrahedron* **2011**, *67*, 5304–5318.
[10] J. Yan, T. Ni, F. Yan, *Tetrahedron Lett.* **2015**, *56*, 1096–1098.
[11] K. Piers, C. Meimaroglou, R. V. Jardine, R. K. Brown, *Can. J. Chem.* **1963**, *41*, 2399–2401
[12] H. Kajita, A. Togni, *ChemistrySelect* **2017**, *2*, 1117–1121.
[13] H. Huang, Z. Zbei, J. Hou, R. Wang, G. Tao, M. Wang, Z. Duan, F. Mathey, *Eur. J. Org. Chem.* **2018**, 2863–2869

reveals that only one bromine atom is incorporated with the consequent formation of **13b**. By warming the reaction up to 0 ºC and performing the reaction for longer time, it was evidenced by ESI-MS analysis that another bromo substitution took place. After 6 h at room temperature, the reaction was stopped because a hexabrominated biindole species was starting to emerge. The reaction was quenched with sat. soln. $Na_2S_2O_3$, diluted with AcOEt (100 mL) and washed successively with sat. soln. of $NaHCO_3$, and brine. Then, after drying the organic solution over Na_2SO_4, it is evaporated at reduced pressure, the crude is triturated with MeOH to afford the insoluble pentabrominated biindole (17 mg, 69% yield, 85% purity). Unfortunately, the ^{1}H NMR of the compound obtained confirmed the formation of the biindole **15** instead of **1**, due to an unexpected regioselective bromination of the position C-6' rather than C-2. The ^{1}H NMR proton signal at C-4' allows easy discrimination between **15** and **1**. This signal resonates at the lowest field among other proton signals due to the anisotropy effect of the nitrile moiety. The appearance of a singlet in **15** confirmed the substitution at C-6', since for **13a** and **13b** this signal appears as a *meta*-coupled doublet (J ~ 2 Hz) as also observed for **1**.

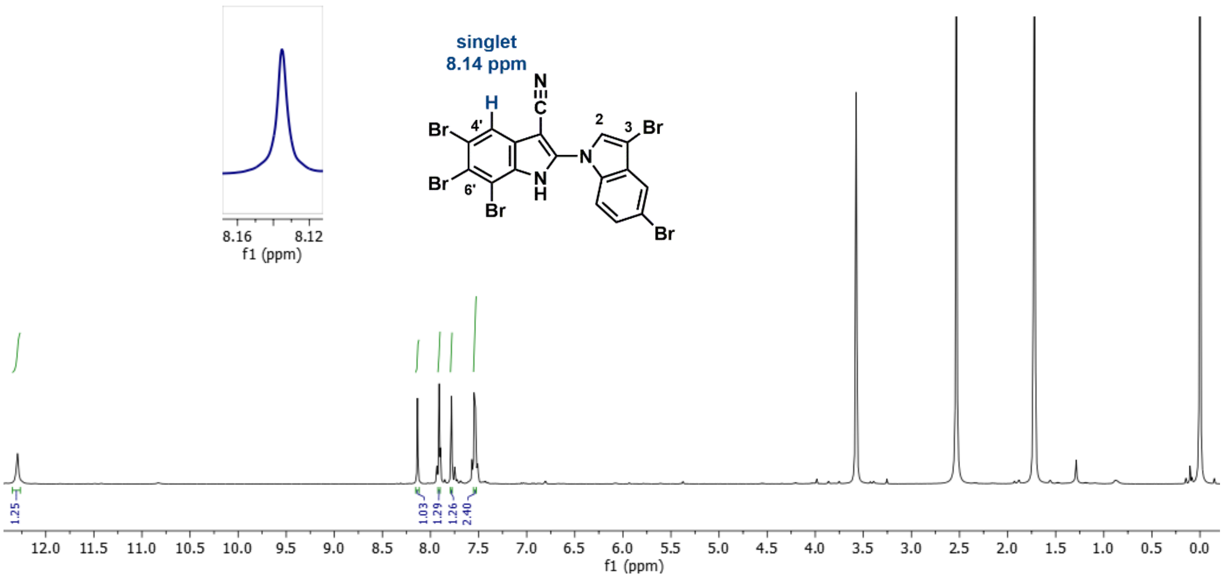

Figure S26. ^{1}H NMR (400 MHz, $CDCl_3$) spectrum of biindole **15**.

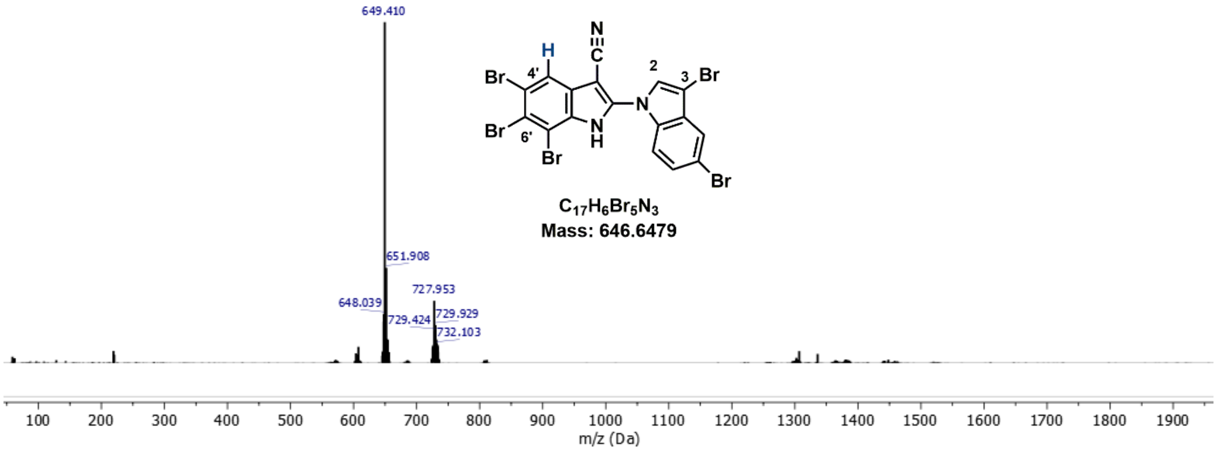

Figure S27. ESI-MS (negative mode) of biindole **15**.

2. Bromination of biindole 16

After concluding that in polar solvents the bromination attempts of **13a** and **13b** failed to generate the natural product, biindole **16** was designed to be a good substrate for the bromination experiments due to the enhanced lipophilicity. The introduction of SEM protecting group enables the study of further bromination reaction in nonpolar solvents (e.g. DCM and DCE). In the following, the different attempts to brominate **16** with $PyHBr_3$, DBDMH, and bromine are described.

Synthesis of biindole 17

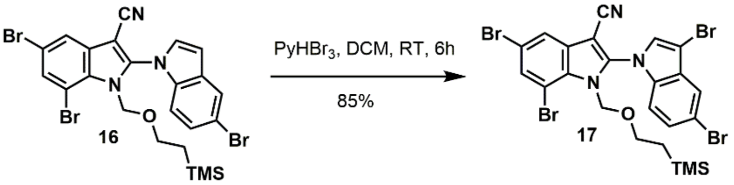

Biindole **16** (100 mg, 0.16 mmol) is dissolved in DCM (10 mL). At room temperature, $PyHBR_3$ (154 mg, 0.48 mmol) is added. The mixture is stirred for 6 h at room temperature, quenched with sat. soln. $Na_2S_2O_3$, diluted with AcOEt (100 mL) and washed successively with sat. soln. of $NaHCO_3$ and brine. After drying the organic solution over Na_2SO_4, it is evaporated at reduced pressure. The crude obtained is purified by column chromatography (n-hex/toluene 3:1) to afford biindole **17** (96 mg, 85%) as a colorless solid. R*f* (toluene/n-hex 1:1) = 0.45. ^{1}H NMR (400 MHz, $CDCl_3$): δ = 7.90 (d, *J* = 1.8 Hz, 1H, H-4'), 7.84 (d, *J* = 1.9 Hz, 1H, H-4), 7.79 (d, *J* = 1.8 Hz, 1H, H-6'), 7.48 (dd, *J* = 8.8, 1.9 Hz, 1H, H-6), 7.43 (s, 1H, H-2), 7.15 (d, *J* = 8.8 Hz, 1H, H-7), 5.74 (d, *J* = 10.4 Hz, 1H, N-CH_2-O), 5.21 (d, *J* = 10.4 Hz, 1H, N-CH_2-O), 3.31 – 3.24 (m, 2H, O-CH_2), 0.82 – 0.74 (m, 2H, CH_2-Si), -0.08 (s, 9H, Si(CH_3)$_3$). ^{13}C NMR (101 MHz, $CDCl_3$): δ = 140.8 (C-2'), 135.2 (C-7a), 133.3 (C-2), 130.1 (C-3a'), 129.8 (C-3a), 129.7 (C-7a'), 128.4 (C-6'), 127.9 (C-6), 123.1 (C-4'), 121.8 (C-4), 117.1 (C-5), 116.5 (C-5'), 112.3 (C-7), 112.0 (CN), 106.2 (C-7'), 96.2 (C-3), 85.1 (C-3'), 72.5 (N-*C*H_2-O), 66.4 (O-*C*H_2), 17.8 (*C*H_2-Si), -1.6 (Si(*C*H_3)$_3$). ESI-MS: m/z = 721.6 $[M+Na]^+$; calcd. for $C_{23}H_{21}{}^{79}Br_4N_3NaOSi$: 721.8.

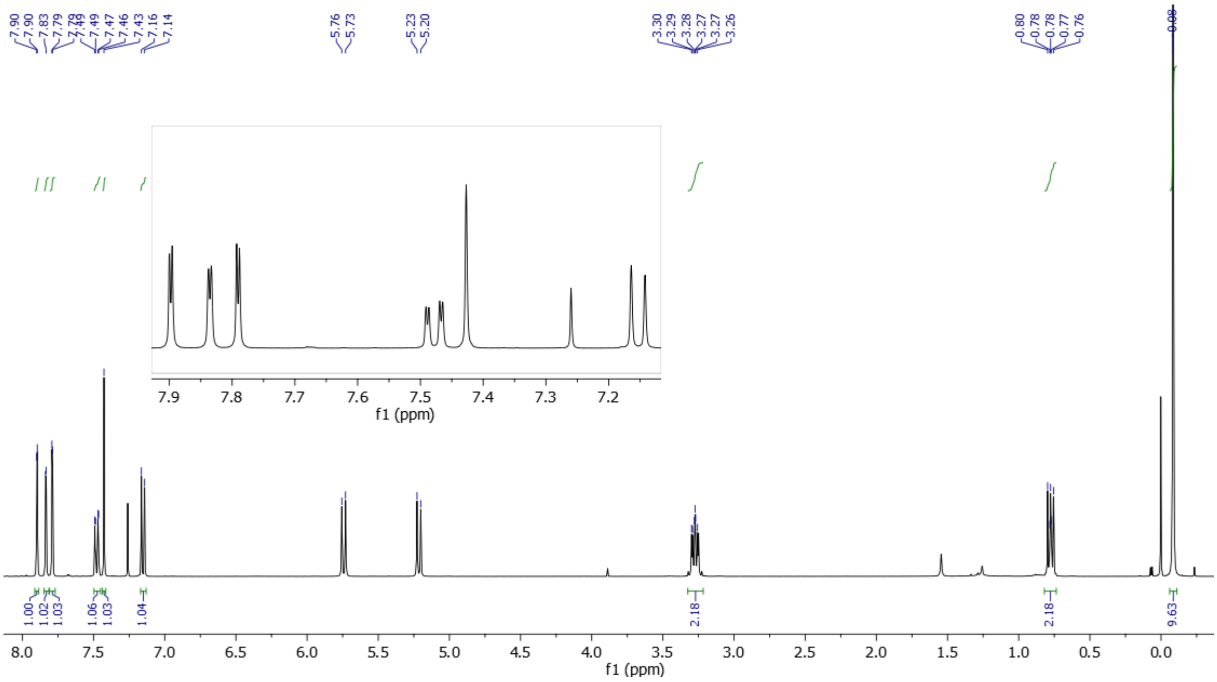

Figure S28. ^{1}H NMR (400 MHz, $CDCl_3$) spectrum of biindole **17**.

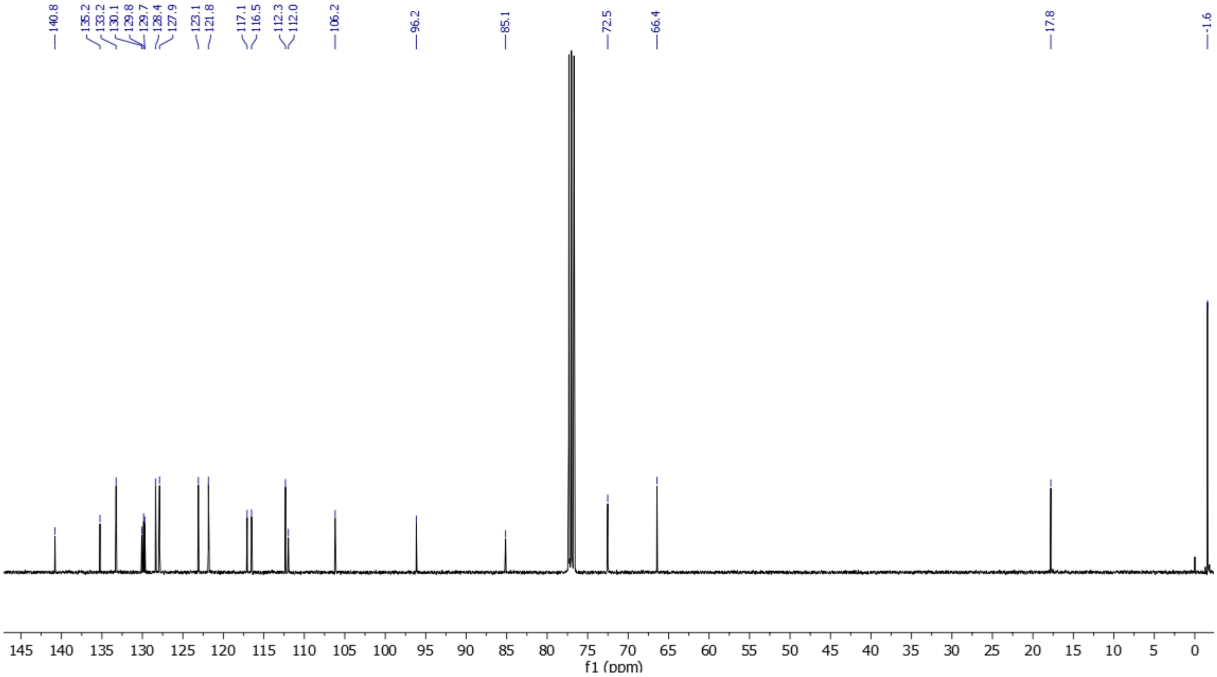

Figure S29. ^{13}C NMR (101 MHz, $CDCl_3$) spectrum of biindole **17**.

Synthesis of biindole 18

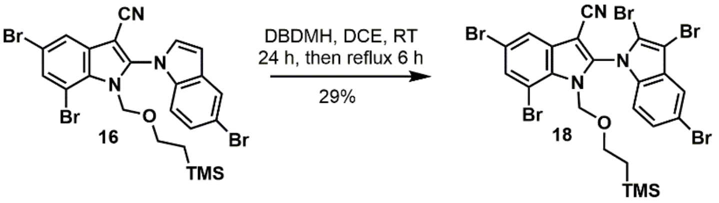

Biindole **16** (100 mg, 0.16 mmol) is dissolved in DCE (10 mL). At room temperature, DBDMH (34 mg, 0.48 mmol) is added. After stirring the reaction mixture at room temperature for 24 h, partial formation of the biindole **18** is detected. Then, the crude is refluxed for 6 h until total consumption of the starting material. The mixture is cooled to room temperature, after which it is quenched with sat. soln. $Na_2S_2O_3$, diluted with AcOEt (100 mL), and washed successively with sat. soln. of $NaHCO_3$ and brine. After drying the organic phase over Na_2SO_4, it is evaporated at reduced pressure. The crude is purified by column chromatography (n-hex/toluene 3:1) to afford biindole **18** in 90% purity (36 mg, 29%) as a colorless solid. R*f* (toluene/n-hex 1:1) = 0.55. ^{1}H NMR (400 MHz, $CDCl_3$): δ = 7.95 (d, *J* = 1.8 Hz, 1H, H-4'), 7.83 (d, *J* = 1.8 Hz, 1H, H-6'), 7.79 (d, *J* = 1.9 Hz, 1H, H-4), 7.41 (dd, *J* = 8.7, 1.9 Hz, 1H, H-6), 6.95 (d, *J* = 8.7 Hz, 1H, H-7), 5.64 (d, *J* = 10.7 Hz, 1H, N-CH_2-O), 5.24 (d, *J* = 10.7 Hz, 1H, N-CH_2-O), 3.23 – 3.04 (m, 2H, O-CH_2), 0.70 – 0.62 (m, 2H, CH_2-Si), -0.15 (s, 9H, Si(CH_3)$_3$). ^{13}C NMR (101 MHz, $CDCl_3$): δ = 138.2 (C-2'), 136.5 (C-7a), 133.8 (C-6'), 130.2 (C-3a'), 129.5 (C-3a), 129.3 (C-7a), 128.4 (C-6), 122.5 (C-4'), 122.2 (C-4), 117.1 (C-5), 116.9 (C-2), 115.7 (C-5'), 112.1 (C-7), 111.6 (CN), 106.3 (C-7'), 98.8 (C-3), 88.6 (C-3'), 72.9 (N-*C*H_2-O), 66.8 (O-*C*H_2), 17.7 (*C*H_2-Si), -1.6 (Si(*C*H_3)$_3$). ESI-MS: m/z = 799.5 $[M+Na]^+$; calcd. for $C_{23}H_{20}{}^{79}Br_5N_3NaOSi$: 799.7. The removal of the SEM protecting group in 50% TFA in DCM quantitatively affords 30 mg (85% purity) of the natural product **1**.

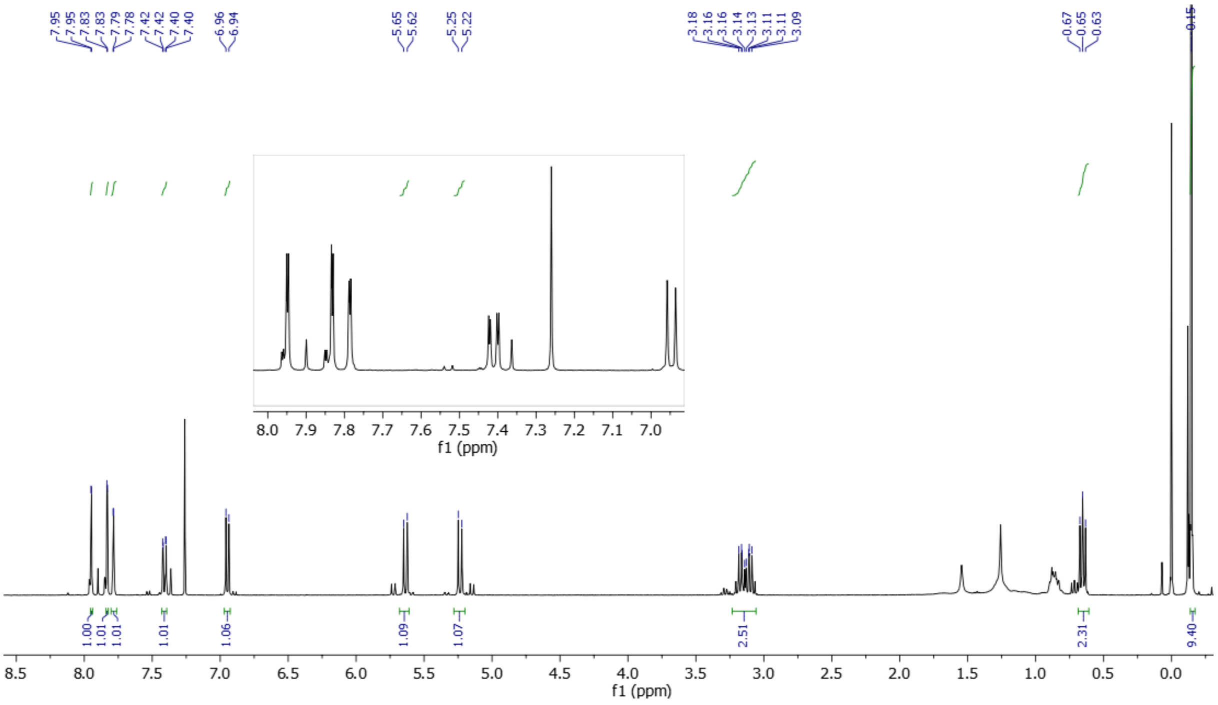

Figure S30. ^{1}H NMR (400 MHz, $CDCl_3$) spectrum of biindole **18**.

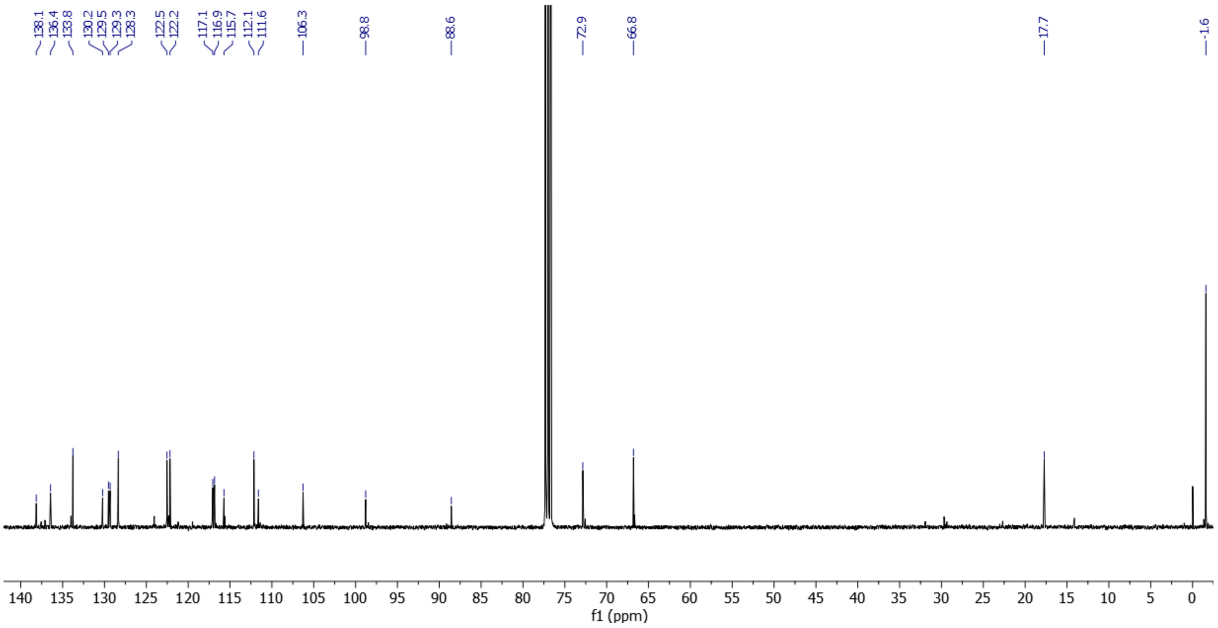

Figure S31. ^{13}C NMR (101 MHz, $CDCl_3$) spectrum of biindole **18**.

Synthesis of AETX (1)

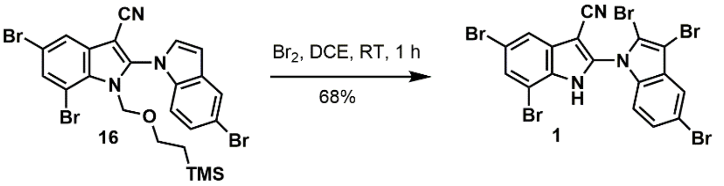

Biindole **16** (100 mg, 0.16 mmol) is dissolved in DCM (10 mL) and bromine (41 µL, 0.8 mmol) is added. The mixture is stirred for 1 h at room temperature, subsequently quenched with soln. aq. of $Na_2S_2O_3$, diluted with AcOEt (100 mL), and washed successively with sat. soln. of $NaHCO_3$ and brine. After drying the organic solution over Na_2SO_4, it is evaporated at reduced pressure. The crude is purified by column chromatography (n-hex/toluene 1:3) to afford the natural product AETX (**1**) (71 mg, 68% yield, 97% purity) as a colorless solid. R*f* (toluene/n-hex 2:1) = 0.45. M.p. = 258-260 ºC. ^{1}H NMR (500 MHz, THF-*d*$_8$): δ = 12.39 (s, 1H, NH), 8.00 (d, *J* = 1.7 Hz, 1H, H-4'), 7.79 (d, *J* = 1.7 Hz, 1H, H-6'), 7.73 (d, *J* = 1.9 Hz, 1H, H-4), 7.43 (dd, *J* = 8.7, 1.9 Hz, 1H, H-6), 7.21 (d, *J* = 8.7 Hz, 1H, H-7). ^{13}C NMR (126 MHz, THF-*d*$_8$): δ = 138.1 (C-2), 137.8 (C-7a), 132.9 (C-3a'), 131.3 (C-6'), 130.2 (C-3a), 129.7 (C-7a'), 129.0 (C-6), 122.7 (C-4'), 122.7 (C-4), 117.4 (C-5), 117.3 (C-2), 116.8 (C-5'), 113.7 (C-7), 112.6 (CN), 107.6 (C-7'), 98.2 (C-3), 88.3 (C-3'). HR-MS: m/z (%)= 645.6417 [M-H]$^-$; calcd. for $C_{17}H_5N_3{}^{79}Br_5$: 645.6406.

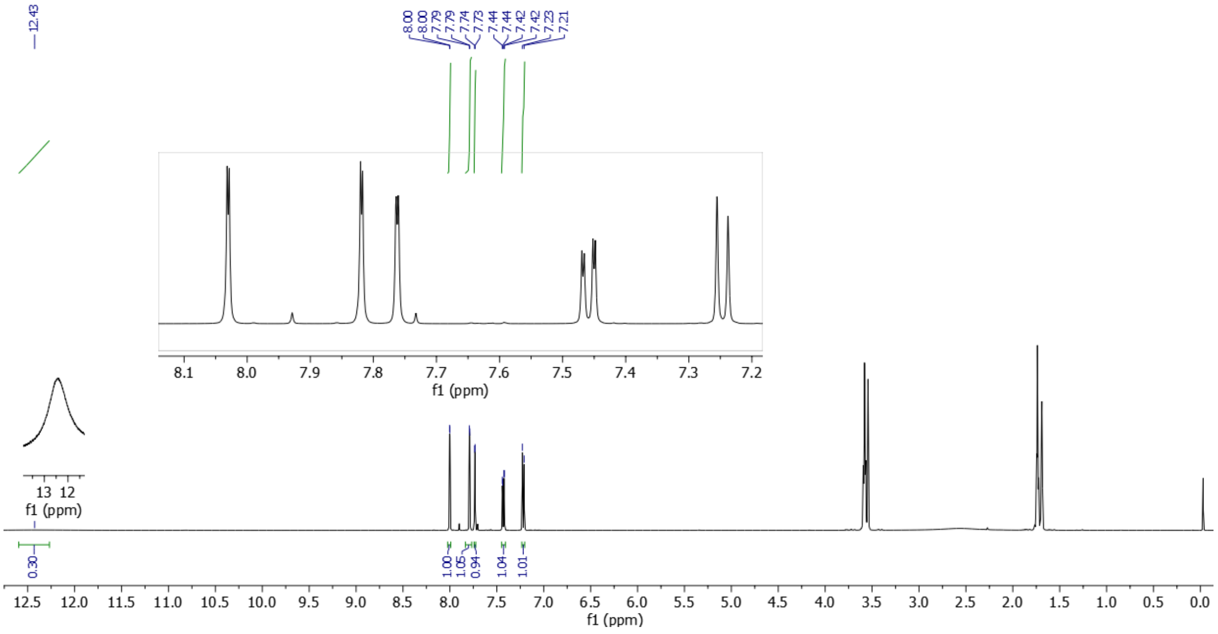

Figure S32. ^{1}H NMR (400 MHz, $CDCl_3$) spectrum of biindole **1** (AETX).

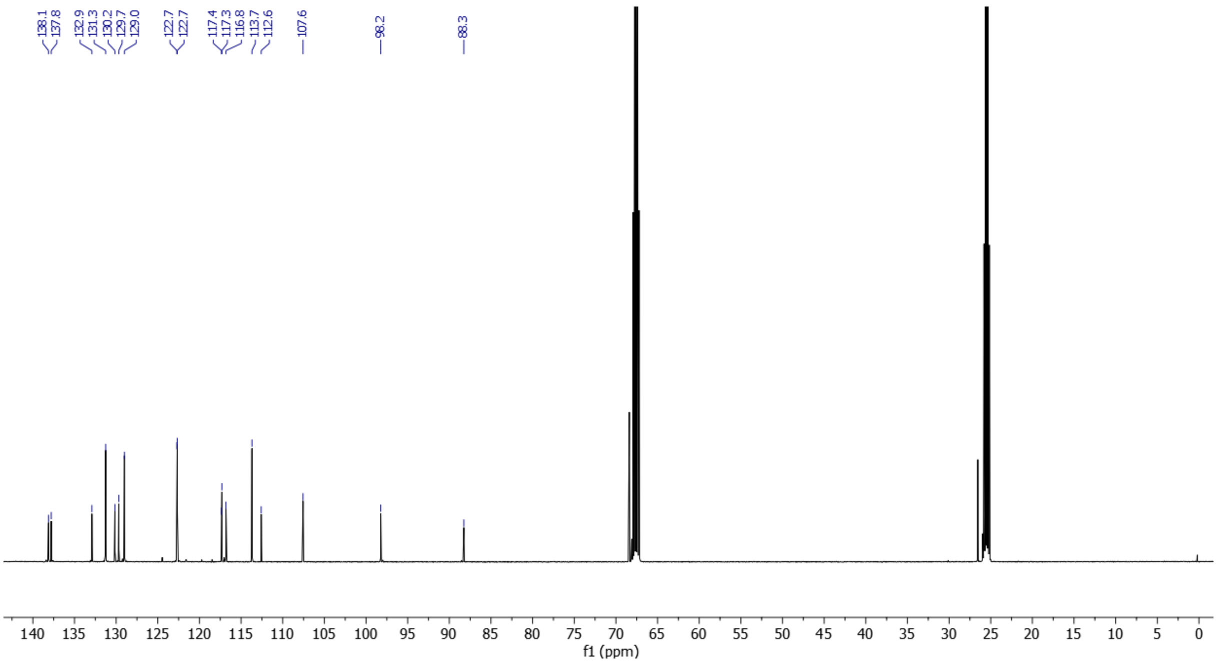

Figure S33. ^{13}C NMR (101 MHz, $CDCl_3$) spectrum of biindole **1** (AETX).

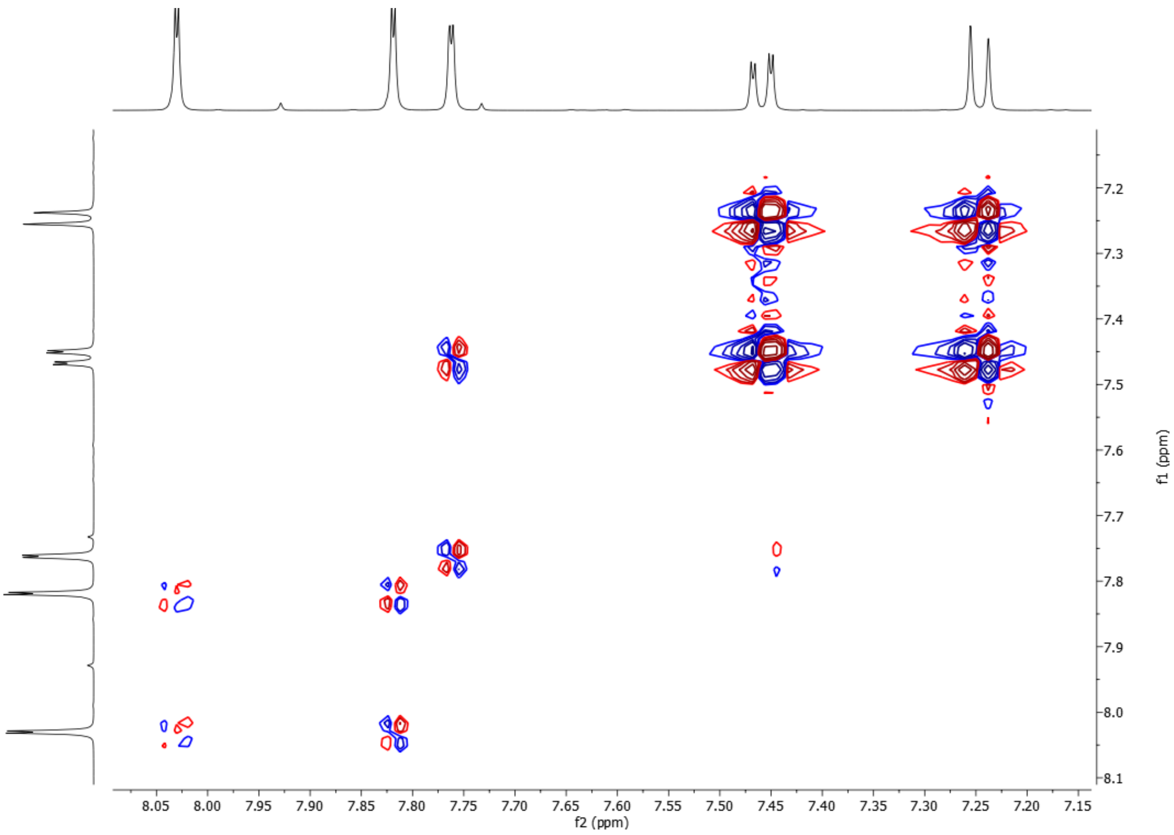

Figure S34. DQF-COSY (400 MHz, $CDCl_3$) spectrum of biindole **1** (AETX).

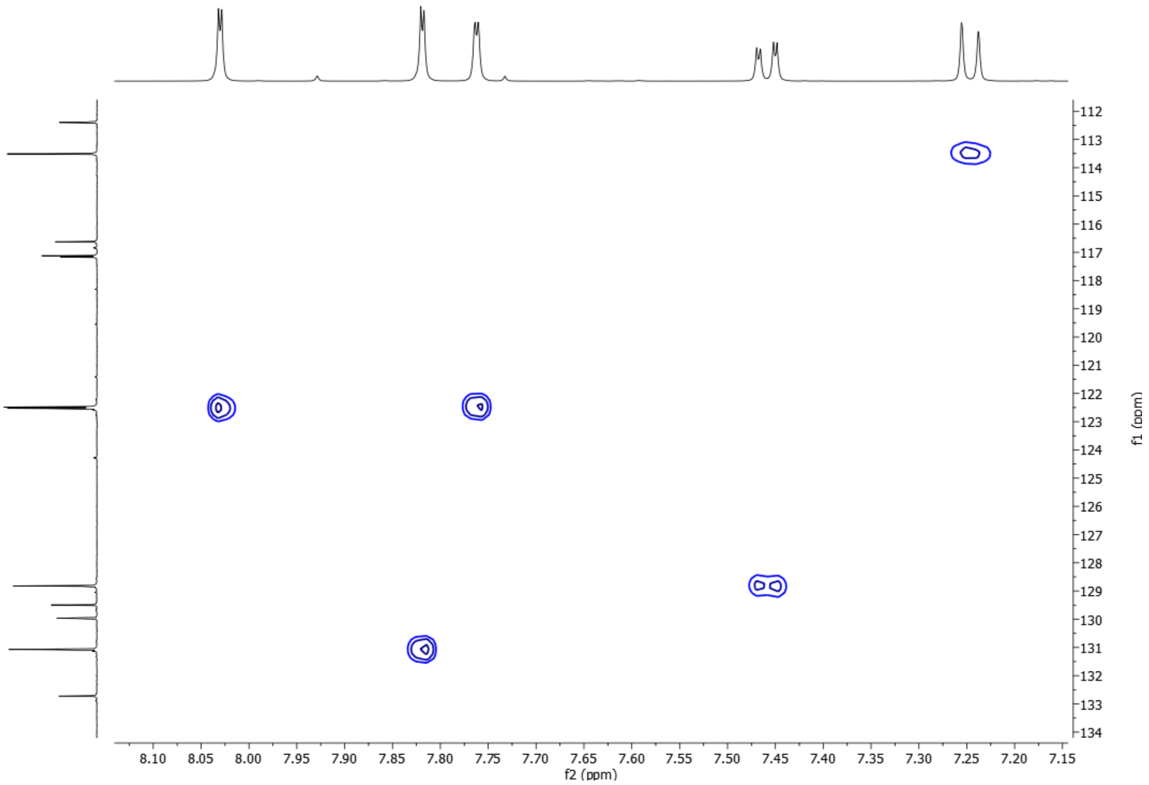

Figure S35. HSQC (400 MHz, $CDCl_3$) spectrum of biindole **1** (AETX).

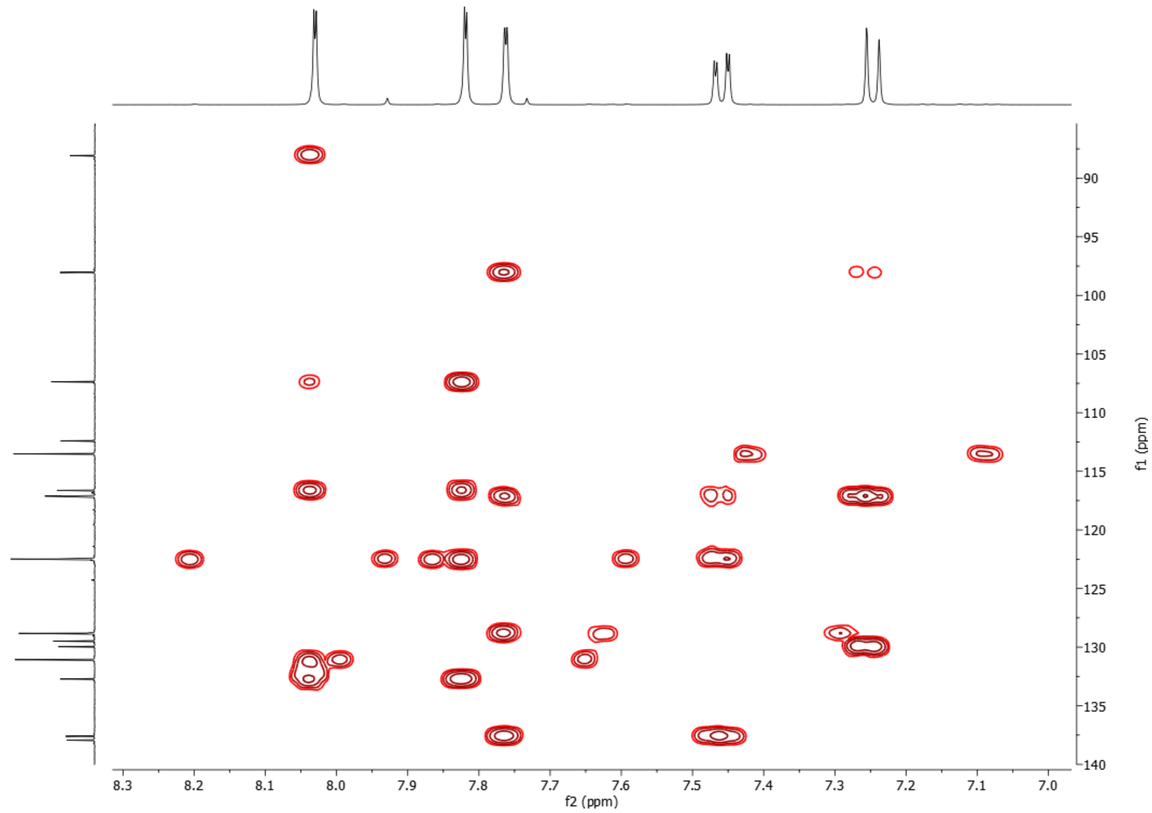

Figure S36. HMBC (400 MHz, $CDCl_3$) spectrum of biindole **1** (AETX).

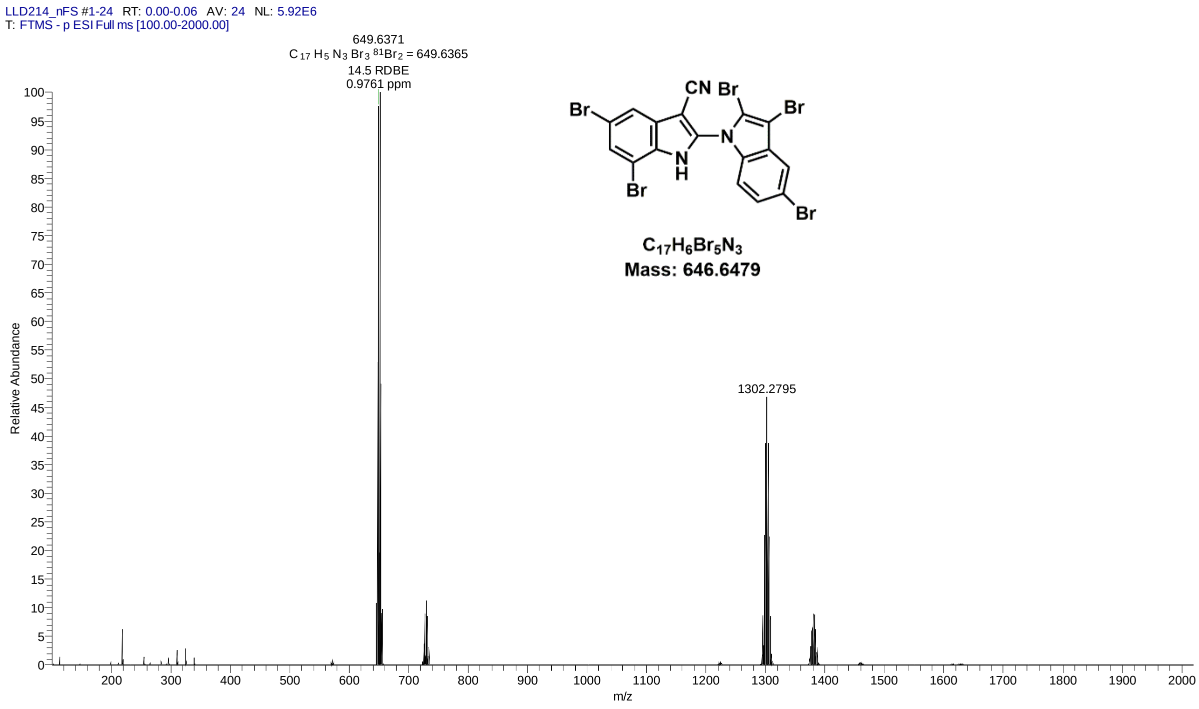

Figure S37. HR-MS (negative mode) of biindole **1** (AETX).
